# Aβ34 is a BACE1-derived degradation intermediate associated with amyloid clearance and Alzheimer’s disease progression

**DOI:** 10.1038/s41467-019-10152-w

**Published:** 2019-05-20

**Authors:** Filip Liebsch, Luka Kulic, Charlotte Teunissen, Adeola Shobo, Irem Ulku, Vivienne Engelschalt, Mark A. Hancock, Wiesje M. van der Flier, Peter Kunach, Pedro Rosa-Neto, Philip Scheltens, Judes Poirier, Paul Saftig, Randall J. Bateman, John Breitner, Christoph Hock, Gerhard Multhaup

**Affiliations:** 1 Department of Pharmacology and Therapeutics and Integrated Program in Neuroscience, McGill University, Montreal, QC H3G 1Y6 Canada; 2 Institute for Regenerative Medicine, University of Zurich, CH-8952 Schlieren, Switzerland; 3 Department of Clinical Chemistry, Amsterdam Neuroscience, Amsterdam UMC, Vrije Universiteit Amsterdam, 1081HZ Amsterdam, The Netherlands; 4 Institut für Chemie und Biochemie, Freie Universität Berlin, 14195 Berlin, Germany; 5 SPR-MS Facility, McGill University, Montreal, QC H3G 1Y6 Canada; 6 Alzheimer Center Amsterdam, Department of Neurology, Amsterdam Neuroscience, Amsterdam UMC, Vrije Universiteit Amsterdam, Amsterdam, 1081HZ The Netherlands; 7 Translational Neuroimaging Laboratory, The McGill University Research Centre for Studies in Aging, Alzheimer’s Disease Research Unit, Douglas Research Institute, McGill University, Montreal, H4H 1R3 QC Canada; 8 Department of Psychiatry, McGill University, Montreal, QC H4H 1R3 Canada; 9 Biochemisches Institut, Christian-Albrechts-Universität-Kiel, 24118 Kiel, Germany; 10 Department of Neurology, Washington University in St. Louis, St. Louis, MO 63110 USA; 11 Neurimmune, CH-8952 Schlieren, Switzerland

**Keywords:** Proteases, Alzheimer's disease

## Abstract

The beta-site APP cleaving enzyme 1 (BACE1) is known primarily for its initial cleavage of the amyloid precursor protein (APP), which ultimately leads to the generation of Aβ peptides. Here, we provide evidence that altered BACE1 levels and activity impact the degradation of Aβ40 and Aβ42 into a common Aβ34 intermediate. Using human cerebrospinal fluid (CSF) samples from the Amsterdam Dementia Cohort, we show that Aβ34 is elevated in individuals with mild cognitive impairment who later progressed to dementia. Furthermore, Aβ34 levels correlate with the overall Aβ clearance rates in amyloid positive individuals. Using CSF samples from the PREVENT-AD cohort (cognitively normal individuals at risk for Alzheimer’s disease), we further demonstrate that the Aβ34/Aβ42 ratio, representing Aβ degradation and cortical deposition, associates with pre-clinical markers of neurodegeneration. We propose that Aβ34 represents a marker of amyloid clearance and may be helpful for the characterization of Aβ turnover in clinical samples.

## Abstract (web summary)

Aβ34 is generated from degradation of Aβ40 and Aβ42 by β-secretase. Here, the authors show that Aβ34 is a marker for amyloid clearance and is elevated in the CSF of patients that go on to convert from mild cognitive impairment to Alzheimer’s disease, suggesting it may be a useful biomarker.

## Introduction

The pathogenesis of Alzheimer’s disease (AD) is characterized by amyloid-β (Aβ) plaque formation in the brain^1,2^. Sequential cleavage of the amyloid precursor protein (APP) by β-secretase (BACE1) and the γ-secretase complex results in the generation of Aβ species of varying lengths, e.g., Aβ38, Aβ40, and Aβ42^3,4,5,6^. More neurotoxic than Aβ38 or Aβ40, the Aβ42 peptide is prone to form oligomers (i.e., precursor to larger fibrils), which are thought to contribute to plaque formation and cognitive decline^7^.

To block the first step of amyloid production, the pharmaceutical industry has focused on inhibitors of BACE1 as a therapeutic strategy for AD. However, BACE1 inhibitors have failed in clinical trials due to side effects, possible toxicity, or the absence of beneficial cognitive outcomes^8^. The lack of success may also relate to the timing of administration, since treatments in the symptomatic stage might be too late.

BACE1 levels are elevated in the neocortex of AD patients^9–11^. Perhaps as a pathological response to fibrillar Aβ, the accumulation of BACE1 in the vicinity of amyloid plaques can enhance local Aβ generation^12–16^. However, excess BACE1 activity can also lead to (i) alternative APP processing at the β’-site, generating metabolically labile Aβ11-X peptides^17^, or (ii) Aβ degradation, by catalyzing the C-terminal truncation of Aβ40 and Aβ42 into non-amyloidogenic Aβ34^18–21^. In human and canine in vivo studies, cerebrospinal fluid (CSF) levels of Aβ34 decline with pharmacological BACE1 inhibition, most likely due to an interruption of the BACE1-mediated degradation of Aβ40 and Aβ42^22,23^. However, the amyloidolytic roles of BACE1 in Aβ metabolism are currently not well defined, either in health (i.e., physiological homeostasis) or disease (i.e., AD pathogenesis).

The pathological cascade of sporadic AD appears to be triggered by impaired Aβ degradation and clearance^24^. Aβ clearance from the brain can occur by several mechanisms including interstitial fluid drainage, cellular uptake, and passive elimination^25,26^. Enzymatic degradation generates specific patterns of soluble Aβ peptides in the CSF^27^, as mediated by Aβ-degrading enzymes (ADEs), which include metalloprotease family members such as endothelin-converting enzyme (ECE), insulin-degrading enzyme (IDE), and neprilysin (NEP)^28^. Results obtained with ^18^O-labeling mass spectrometry demonstrated that the Aβ peptide pattern in CSF is not generated by proteolytic activities in CSF itself—except in the acute phase of a bacterial meningitis^29^—but Aβ fragments are likely generated prior to entering the CSF.

The normal clearance rate for Aβ40 or Aβ42 in human CSF is estimated to be ~8% per hour^30^, but clearance is impaired by approximately 30% in AD patients^24^. Aβ stable isotope labeling kinetics (SILK) studies found that production and clearance of soluble Aβ isoforms are similar for Aβ38 and Aβ40, but Aβ42 turnover is altered with increasing age and amyloidosis^31^.

Since we have previously shown that Aβ34 is a common intermediate in the enzymatic processing of two distinct Aβ degradation pathways^6^, the present study examines the levels and metabolism of Aβ34 in the brains of BACE1-deficient mice, in brain and CSF of rats treated with a BACE1-specific inhibitor, a cultured human neuronal cell line (SH-SY5Y), and CSF samples from individuals at various clinical stages of AD. To accomplish this, we utilize a custom, ultra-sensitive Meso Scale Discovery (MSD) electrochemiluminescence assay, using a monoclonal neo-epitope antibody that binds specifically to the C-terminus of Aβ34 with nanomolar affinity. Our results show that cerebral BACE1 levels are limiting for Aβ34 generation in vivo. Specifically, in well-characterized clinical groups, CSF levels of Aβ34 are notably elevated in individuals with mild cognitive impairment (MCI) who later progressed to AD dementia. Compared with the classical Aβ40/Aβ42 ratio (i.e., marker of amyloid deposition in clinical practice), the Aβ34/Aβ42 ratio improves our ability to distinguish between individuals with MCI who later converted to AD from those who did not. Among cognitively normal individuals at risk for AD, an elevated CSF-Aβ34/Aβ42 ratio is detected together with current biomarkers of pre-clinical AD, such as elevated CSF levels of total-tau (t-tau) and phosphorylated (P_181_)-tau. Furthermore, the overall Aβ clearance rates positively correlate with CSF-Aβ34 levels in amyloid-positive (Aβ+) individuals.

While the interesting Aβ34 biology of this article directly impacts the design of future studies looking at Aβ turnover and clinical studies involving BACE inhibitors, we anticipate that combining markers of amyloid clearance (Aβ34 measurements) and deposition (well-established Aβ42 measurements in CSF) may provide a more complete biomarker panel to assess AD samples (i.e., early-stage biochemical changes vs. late-stage plaque/tangle pathology). Future studies are needed to validate whether an increased CSF-Aβ34/Aβ42 ratio may provide an opportunity for earlier intervention strategies.

## Results

### Cerebral Aβ34 is decreased in BACE1-deficient mice

**Fig. 1:**
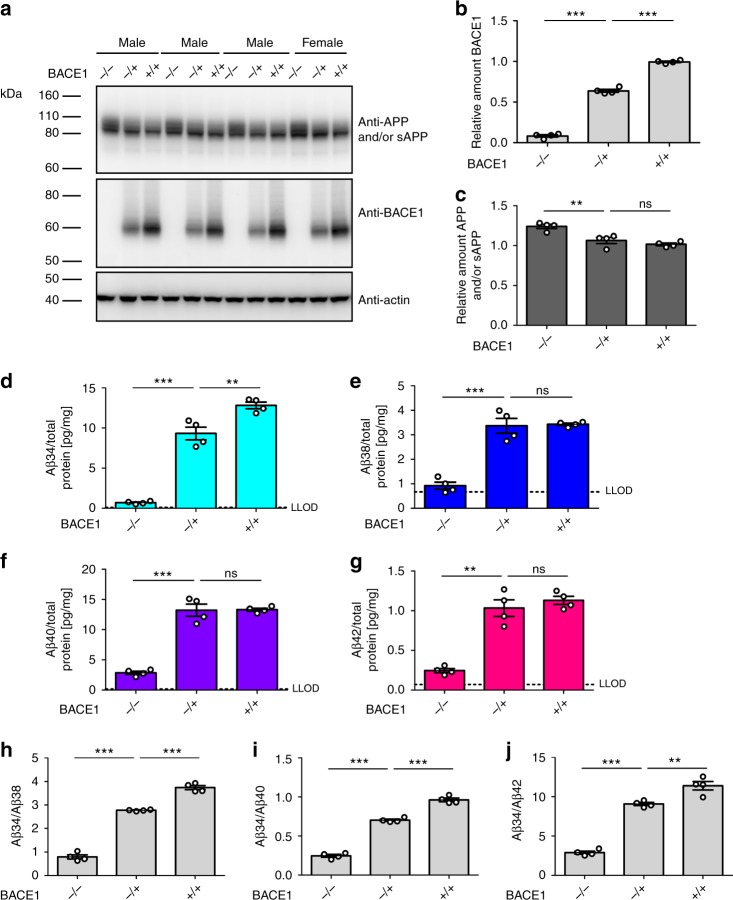
Endogenous BACE1 generates Aβ34 in the murine brain. Endogenous levels of murine APP and/or sAPP, BACE1, and Aβ in BACE1−/−, BACE1+/−, and wild-type littermates (+/+). *N* = 4 animals per group. Western blot of endogenous APP and/or sAPP and BACE1 expression in male and female mice (**a**) and corresponding quantification of relative protein amounts of BACE1 (**b**) and APP and/or sAPP (**c**). Quantification (pg/mg total protein) of absolute amounts of Aβ34 (**d**), Aβ38 (**e**), Aβ40 (**f**), and Aβ42 (**g**) as determined by custom 4-plex MSD multiplexing assays. Ratios of Aβ34/Aβ38 (**h**), Aβ34/Aβ40 (**i**), and Aβ34/Aβ42 (**j**) are displayed. Statistics: **b** one-way ANOVA, *F*(2, 9) = 1021, *p* < 0.0001, **c** one-way ANOVA, *F*(2, 9) = 17.28, *p* < 0.001, **d** one-way ANOVA, *F*(2, 9) = 145.7, *p* < 0.0001, **e** one-way ANOVA, *F*(2, 9) = 53.68, *p* < 0.0001, **f** one-way ANOVA, *F*(2, 9) = 95.65, *p* < 0.0001, **g** one-way ANOVA, *F*(2, 9) = 49.70, *p* < 0.0001, **h** one-way ANOVA, *F*(2, 9) = 425.2, *p* < 0.0001, **i** one-way ANOVA, *F*(2, 9) = 378.0, *p* < 0.0001, **j** one-way ANOVA, *F*(2, 9) = 157.6, *p* < 0.0001. Bars and error bars indicate mean ± s.e.m. Tukey’s post-hoc tests were performed for pairwise comparisons; selected comparisons are highlighted ***p < 0.001, **p < 0.01, ns = nonsignificant *p* > 0.05. For each target, the MSD software computes the lower limit of detection (LLOD) as 2.5 standard deviations above the blank

Given that BACE1 can directly cleave longer Aβ species between Leu34 and Met35 in vitro^19,20^ (Supplementary Fig. 1), we hypothesized that Aβ degradation would be affected by altered BACE1 expression levels in vivo. To test this, we analyzed endogenous cerebral BACE1, APP, and Aβ levels from BACE1−/− and BACE1+/− mice, as well as their wild-type (BACE1+/+) littermates (Fig. 1a–g). In agreement with previously published results^32,33^, BACE1 protein levels in the brain of BACE1+/− mice were approximately half that of wild-type mice, as analyzed by western blot (Fig. 1a, b). Furthermore, BACE1−/− mice had significantly elevated levels of cerebral APP and/or soluble APP (sAPP) compared with their BACE1+/− and wild-type littermates, whereas there was no significant difference between BACE1+/− and wild-type animals (Fig. 1a, c). Notably, the former is possibly due to increased levels of full-length APP^34^.

We next measured endogenous levels of Aβ34, Aβ38, Aβ40, and Aβ42 peptides in the brains of these same mice using a custom 4-plex MSD multiplexing assay (Supplementary Methods, Supplementary Figs. 2, 3). In agreement with previous findings in mice and rats^32,35,36^, Aβ38, Aβ40, and Aβ42 were significantly decreased in BACE1−/− mouse brains, whereas their levels did not differ significantly between BACE1+/− and wild-type littermates (Fig. 1e–g). As expected for a fragment generated by BACE1 activity, cerebral Aβ34 levels were decreased in BACE1−/− mice compared with BACE1+/− (Fig. 1d). Notably, Aβ34 levels were also significantly lower in BACE1+/− mice compared with wild-type littermates. Apparently, the cerebral amyloidogenic processing of endogenous APP by BACE1 is not impaired by a 50% decreased enzyme availability, as it is indistinguishable in heterozygous knockouts and wild-type littermates (Fig. 1e–g). Furthermore, the Aβ34/Aβ38, Aβ34/Aβ40, and Aβ34/Aβ42 ratios revealed a step-wise decrease with decreasing BACE1 levels (Fig. 1h–j). These findings support the concept that cerebral BACE1 levels are limiting for its amyloidolytic activity in vivo relying on conversion of the longer Aβ species to Aβ34, whereas BACE1 levels are not limiting for APP cleavage resulting in Aβ38, Aβ40, and Aβ42.

### BACE1 inhibition lowers rat CSF and brain Aβ34 levels

**Fig. 2:**
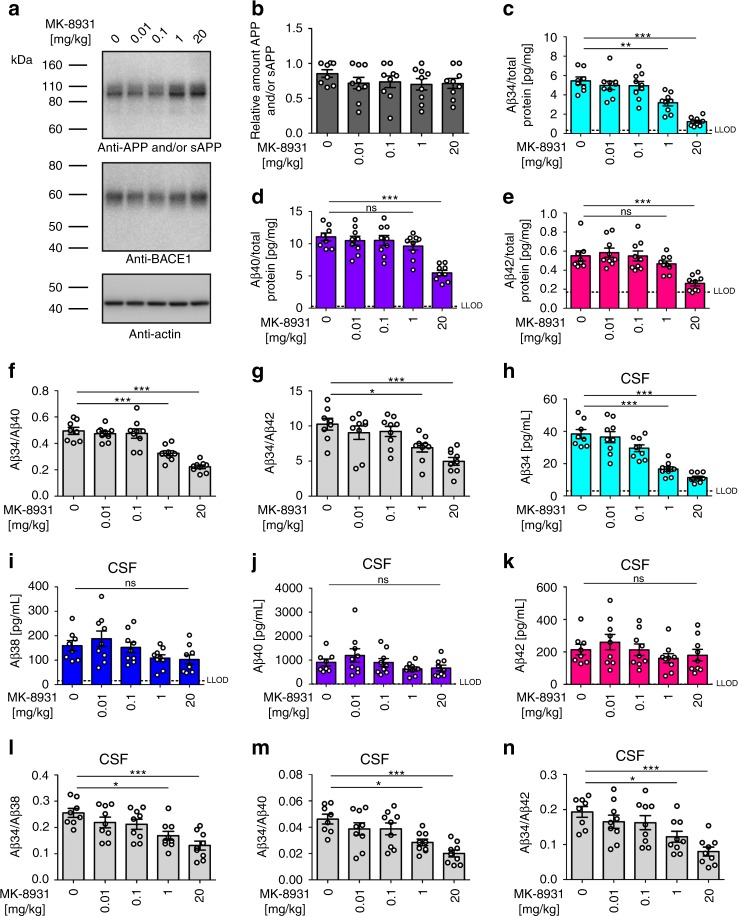
Pharmacological inhibition of Aβ34 generation in rats. Endogenous levels of APP and/or sAPP, BACE1, and Aβ in rats 1 h after intravenous injection of the indicated concentrations (mg/kg) of the BACE1-specific inhibitor (MK-8931) *n* = 9 animals per group or vehicle *n* = 8 animals. Western blot of endogenous APP and/or sAPP and BACE1 expression (**a**) and corresponding quantification of relative protein amounts of APP and/or sAPP (**b**). Quantification (pg/mg total protein) of absolute amounts of Aβ34 (**c**), Aβ40 (**d**), and Aβ42 (**e**) as determined by custom MSD multiplexing assays. Ratios of Aβ34/Aβ40 (**f**) and Aβ34/Aβ2 (**g**) are displayed. Quantification of CSF levels of Aβ34 (**h**), Aβ38 (**i**), Aβ40 (**j**), and Aβ42 (**k**), as well as display of the ratios Aβ34/Aβ38 (**l**), Aβ34/Aβ40 (**m**), and Aβ34/Aβ42 (**n**). Statistics: **b** one-way ANOVA, *F*(4, 39) = 0.63, *p* > 0.05, **c** one-way ANOVA, *F*(4, 39) = 22.40, *p* < 0.0001, **d** one-way ANOVA, *F*(4, 39) = 12.81, *p* < 0.0001, **e** one-way ANOVA, *F*(4, 39) = 9.38, *p* < 0.0001, **f** one-way ANOVA, *F*(4, 39) = 25.93, *p* < 0.0001, **g** one-way ANOVA, *F*(4, 39) = 8.02, *p* < 0.0001, **h** one-way ANOVA, *F*(4, 39) = 26.55, *p* < 0.0001, **i** one-way ANOVA, *F*(4, 39) = 2.69, *p* < 0.05, **j** one-way ANOVA, *F*(4, 39) = 1.71, *p* > 0.05, **k** one-way ANOVA, *F*(4, 39) = 1.09, *p* > 0.05, **l** one-way ANOVA, *F*(4, 39) = 6.87, *p* < 0.001, **m** one-way ANOVA, *F*(4, 39) = 7.36, *p* < 0.001, **n** one-way ANOVA, *F*(4, 39) = 6.74, *p* < 0.001. Bars and error bars indicate mean ± s.e.m. Tukey’s post-hoc tests were performed for pairwise comparisons; selected comparisons are highlighted ***p < 0.001, **p < 0.01, *p < 0.05, ns = nonsignificant *p* > 0.05

To independently assess whether the above findings hold true for the pharmacological intervention with BACE1 activity, wild-type rats were treated with the BACE1-specific inhibitor MK-8931^37^. The inhibitor had no effect on cerebral APP and/or sAPP levels (Fig. 2a, b). Cerebral Aβ34 levels were significantly decreased in rats treated with concentrations of 1 and 20 mg/kg (Fig. 2c). In contrast, cerebral Aβ40 and Aβ42 levels were only decreased in the 20 mg/kg but not the 1 mg/kg cohort (Fig. 2d, e). Consequently, the ratios Aβ34/Aβ40 and Aβ34/Aβ42 were significantly decreased at 1 and 20 mg/kg (Fig. 2f, g). In agreement with our findings in BACE1+/− mice, these results indicate that cerebral Aβ34 levels are more sensitive to changes in BACE1 activity than the longer Aβ species.

We next measured CSF levels of Aβ34, Aβ38, Aβ40, and Aβ42. CSF-Aβ34 was significantly decreased in the cohorts treated with 1 and 20 mg/kg (Fig. 2h), however, there was no change for Aβ38, Aβ40, and Aβ42 (Fig. 2i–k). Furthermore, the ratios Aβ34/Aβ38, Aβ34/Aβ40, and Aβ34/Aβ42 in CSF were significantly decreased in the animals treated with 1 and 20 mg/kg (Fig. 2l–n). Overall, the results of these experiments demonstrate that the pharmacological inhibition of BACE1 differentially affects the amyloidogenic and the amyloidolytic activities of the enzyme in vivo. Importantly, the latter seems more sensitive than the former to the amount (Fig. 1) and activity (Fig. 2) of BACE1.

### Aβ34 is an Aβ degradation intermediate

**Fig. 3:**
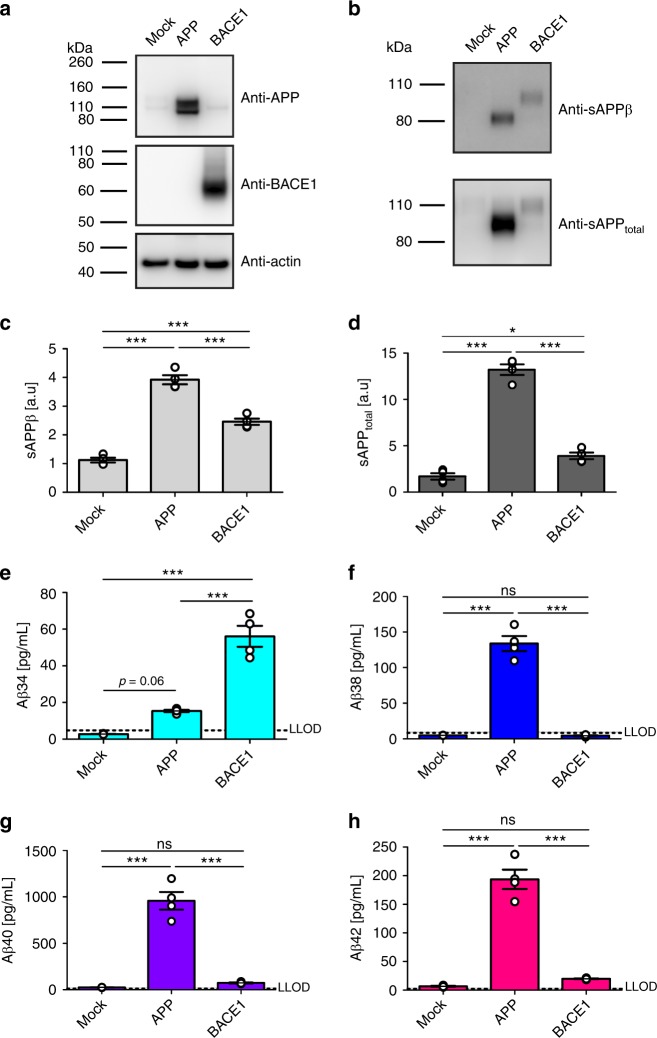
Surplus of APP or BACE1 differentially affect APP processing. Using SH-SY5Y cells stably expressing APP695 or BACE1, cleavage of APP was analyzed by western blot and ultra-sensitive MSD assay. Representative western blots for the examination of APP, BACE1, sAPPβ, and sAPP_total_ (**a**, **b**), and the corresponding quantification for the relative amounts of sAPPβ (**c**) and sAPP_total_ (**d**). MSD multiplexing to quantify the absolute amounts of Aβ34 (**e**), Aβ38 (**f**), Aβ40 (**g**), and Aβ42 (**h**). Data were collected from four independent experiments. Bars and error bars indicate mean ± s.e.m. **c–h** Data were analyzed with one-way ANOVAs and Tukey’s post-hoc tests were performed for pairwise comparisons; selected comparisons are highlighted ***p < 0.001, *p < 0.05, ns = nonsignificant *p* > 0.05. **c** sAPPβ, *F*(2, 9) = 131.2, *p* < 0.0001, **d** sAPP_total_, *F*(2, 9) = 190.3, *p* < 0.0001, **e** Aβ34, *F*(2, 9) = 70.04, *p* < 0.0001, **f** Aβ38, *F*(2, 9) = 149.0, *p* < 0.0001, **g** Aβ40, *F*(2, 9) = 89.96, *p* < 0.0001, **h** Aβ42, *F*(2, 9) = 113.6, *p* < 0.0001

Previous studies showed that APP and BACE1 expression can change the quantity of Aβ produced^33,38^. Therefore, we tested whether Aβ34 levels would be affected by a surplus of either substrate or enzyme in a human neuronal cell type. Using human neuroblastoma (SH-SY5Y) cells stably overexpressing APP or BACE1 (Fig. 3a), we measured secreted levels of Aβ34, Aβ38, Aβ40, and Aβ42 in the supernatants using the ultra-sensitive 4-plex assay, whereas sAPPβ and sAPP_total_ were detected by western blot (Fig. 3a–h). Overexpression of APP mildly increased Aβ34 levels (not statistically different from empty plasmid (Mock)-transfected cells, *p* = 0.06) and significantly elevated sAPPβ, sAPP_total_, Aβ38, Aβ40, and Aβ42, as compared with Mock-transfected cells (Fig. 3b–h). Conversely, in BACE1-overexpressing cells, Aβ34 levels were significantly elevated approximately threefold compared with APP-overexpressing conditions, whereas Aβ38, Aβ40, and Aβ42 levels showed only a mild trend toward elevation compared with the control (Fig. 3e–h). Furthermore, more APP was shed from either of these cell lines (Fig. 3c, d). Note that the apparent molecular weights of endogenous APP and sAPPs are slightly higher than those from APP695-transfected cells (Fig. 3a, b), which is likely due to the fact that undifferentiated SH-SY5Y cells express not only APP695 but also splice variants with KPI- and OX-2 domains^39,40^. Overall, we conclude that a surplus of either substrate or enzyme affects APP processing differentially in the human neuronal cell line, resulting in (i) increased Aβ38, Aβ40, and Aβ42 release from APP-overexpressing cells, or (ii) an enhanced degradation of all longer Aβ forms into Aβ34 in BACE1-overexpressing cells.

**Fig. 4:**
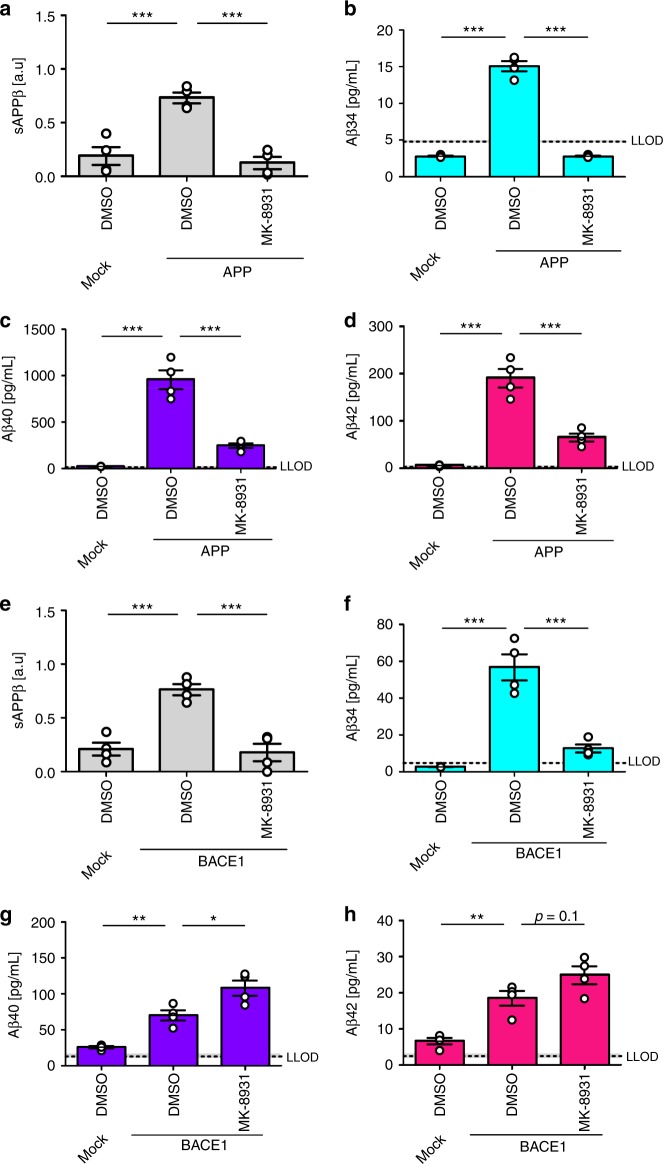
Surplus of APP or BACE1 affect BACE1 inhibition. Cleavage of APP was analyzed by western blot and ultra-sensitive MSD assays. Absolute or relative amounts of products were quantified from SH-SY5Y cells stably expressing APP695 (**a**–**d**) or BACE1 (**e**–**h**). Quantification of relative amounts of sAPPβ (**a**, **e**), and absolute amounts of Aβ34 (**b**, **f**), Aβ40 (**c**, **g**), and Aβ42 (**d**, **h**). Data were collected from four independent experiments. Bars and error bars indicate mean ± s.e.m. Tukey’s post-hoc tests were performed for pairwise comparisons; selected comparisons are highlighted ***p < 0.001, **p < 0.01, *p < 0.05. **a** sAPPβ, one-way ANOVA, *F*(2, 9) = 26.6, *p* < 0.001, **b** Aβ34, one-way ANOVA, *F*(2, 9) = 296.1, *p* < 0.0001, **c** Aβ40, one-way ANOVA, *F*(2, 9) = 65.9, *p* < 0.0001, **d** Aβ42, one-way ANOVA, *F*(2,9) = 59.4, *p* < 0.0001, **e** sAPPβ, one-way ANOVA, *F*(2, 9) = 25.4, *p* < 0.001, **f** Aβ34, one-way ANOVA, *F*(2,9) = 45.3, *p* < 0.0001, **g** Aβ40, one-wayANOVA, *F*(2, 9) = 30.8, *p* < 0.0001, **h** Aβ42, one-way ANOVA, *F*(2, 9) = 23.3, *p* < 0.001

To investigate whether BACE1 inhibition differentially affects the Aβ profiles, SH-SY5Y cells overexpressing APP or BACE1 were treated with MK-8931^37^ at approximately 20-fold of its published IC_50_ (i.e., [MK-8931] = 100 nM, Fig. 4, Supplementary Fig. 4). Conditioned medium from APP-overexpressing SH-SY5Y cells showed significantly decreased levels of sAPPβ, Aβ34, Aβ38, Aβ40, and Aβ42 in the presence of MK-8931 (Fig. 4a–d, Supplementary Fig. 4a–c). Furthermore, a dose-inhibition curve for MK-8931 was performed and sAPPβ (western blot), Aβ34 (MSD 1-plex), and Aβ40 (MSD 1-plex) were measured (Supplementary Fig. 4d–f). In APP-overexpressing SH-SY5Y cells, MK-8931 dose-dependently decreased sAPPβ (pIC_50_ = 10.7 ± 0.3 (s.e.m.)), Aβ34 (pIC_50_ = 14.1 ± 0.6), and Aβ40 levels (pIC_50_ = 11.3 ± 0.7) (Supplementary Fig. 4d–f). In contrast, in conditioned medium from BACE1-overexpressing SH-SY5Y cells, only the levels of sAPPβ and Aβ34 were significantly decreased in the presence of the inhibitor (Fig. 4e, f, Supplementary Fig. 4g, h), whereas Aβ38 and Aβ40 were significantly increased and Aβ42 levels showed a trend for being elevated, *p* = 0.1 (Supplementary Fig. 4i, Fig. 4g, h). In BACE1-overexpressing SH-SY5Y cells, MK-8931 only dose-dependently decreased sAPPβ (pIC_50_ = 8.3 ± 0.3) and Aβ34 levels (pIC_50_ = 7.3 ± 0.2) (Supplementary Fig. 4j, l). Interestingly, Aβ40 levels were decreased at high inhibitor concentrations (p[MK-8931] > −6), elevated at intermediate concentrations (−10 < p[MK-8931] < −6) and unchanged at low concentrations (−10 > p[MK-8931]) (Supplementary Fig. 4k). Ultimately, these results show that the response to pharmacological BACE1 inhibition depends on the relative abundances of BACE1 and its substrate APP. Notably, inhibition with insufficient amounts of compound at high levels of BACE1 could result in undesired elevated Aβ40 and Aβ42 levels. However, BACE1 inhibitor doses used in vivo can effectively reduce the levels of longer Aβ species^37^.

**Fig. 5:**
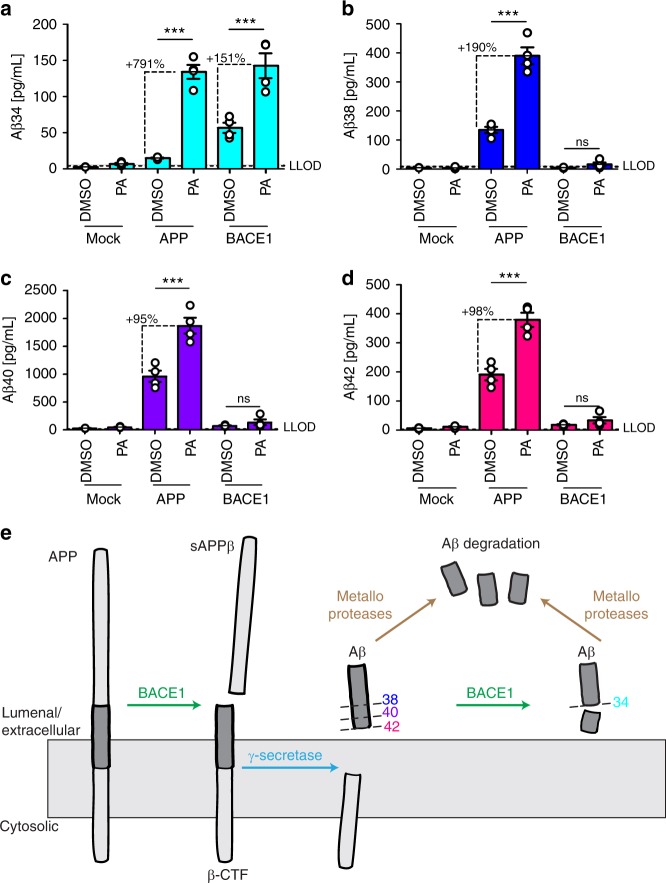
Aβ34 degradation by PA-sensitive metalloproteases. Using SH-SY5Y cells stably expressing APP695 or BACE1, MSD multiplexing to quantify the absolute amounts of Aβ34 (**a**), Aβ38 (**b**), Aβ40 (**c**), and Aβ42 (**d**) was performed. Data were collected from four independent experiments. Bars and error bars indicate mean ± s.e.m. Data were analyzed with two-way ANOVAs and significant interactions were followed up with simple main effects @treatment. ***p < 0.001, ns = nonsignificant *p* > 0.05. **a** Aβ34, interaction *F*(2, 18) = 23.93, *p* < 0.0001, simple main effects @Mock *F*(1, 18) = 0.12, *p* > 0.05, @APP *F*(1, 18) = 97.02, *p* < 0.0001, @BACE1 *F*(1, 18) = 50.41, *p* < 0.0001, **b** Aβ38, interaction *F*(2, 18) = 63.26, *p* < 0.0001, simple main effects @Mock *F*(1, 18) = 0.002, *p* > 0.05, @APP *F*(1, 18) = 197.54, *p* < 0.0001, @BACE1 *F*(1, 18) = 0.39, *p* > 0.05, **c** Aβ40, interaction *F*(2, 18) = 25.54, *p* < 0.0001, simple main effects @Mock *F*(1, 18) = 0.03, *p* > 0.05, @APP *F*(1, 18) = 73.97, *p* < 0.0001, @BACE1 *F*(1, 18) = 0.34, *p* > 0.05, **d** Aβ42, interaction *F*(2, 18) = 29.08, *p* < 0.0001, simple main effects @Mock *F*(1, 18) = 0.07, *p* > 0.05, @APP *F*(1, 18) = 97.47, *p* < 0.0001, @BACE1 *F*(1, 18) = 0.67, *p* > 0.05. Schematic model (**e**) describes the proposed APP and Aβ processing pathways involving BACE1 and metalloproteases

ADEs include the metalloproteases, a large group of proteases that likely cleave Aβ peptide substrates^28^. In general, ADE-derived fragments are short, soluble, and not prone to aggregate. To investigate how Aβ34, Aβ38, Aβ40, and Aβ42 degradation is affected by metalloproteases, we treated APP- or BACE1-overexpressing SH-SY5Y cells with the metalloprotease inhibitor phosphoramidon (PA). Supernatants were analyzed using the 4-plex MSD assay as before (Fig. 5). In APP-overexpressing cells, PA treatment significantly elevated the levels of all Aβ species measured (Fig. 5a–d). In the presence of PA, however, Aβ34 was increased approximately ninefold, whereas the levels of Aβ38, Aβ40, and Aβ42 were only about two to threefold higher (Fig. 5a–d). In agreement with previously published data^21^, these findings imply that Aβ34 is more sensitive to metalloprotease-mediated degradation compared with the longer species tested. In BACE1-overexpressing cells, Aβ34 levels were increased by about 2.5-fold in the presence of PA, whereas Aβ38, Aβ40, and Aβ42 levels were not significantly changed (Fig. 5a–d). Together, these results suggest that Aβ34 is a stable Aβ degradation intermediate of the amyloid degradation cascade. Thus, we propose that in the presence of surplus APP substrate, Aβ38, Aβ40, and Aβ42 are degraded by both metalloproteases and the BACE1-mediated Aβ34 pathway (Fig. 5e). Conversely, in the presence of surplus BACE1 enzyme, longer forms of Aβ are predominantly degraded by BACE1, yielding Aβ34 as a metastable cleavage product (Fig. 5e).

### CSF-Aβ34 levels are associated with AD progression

**Fig. 6:**
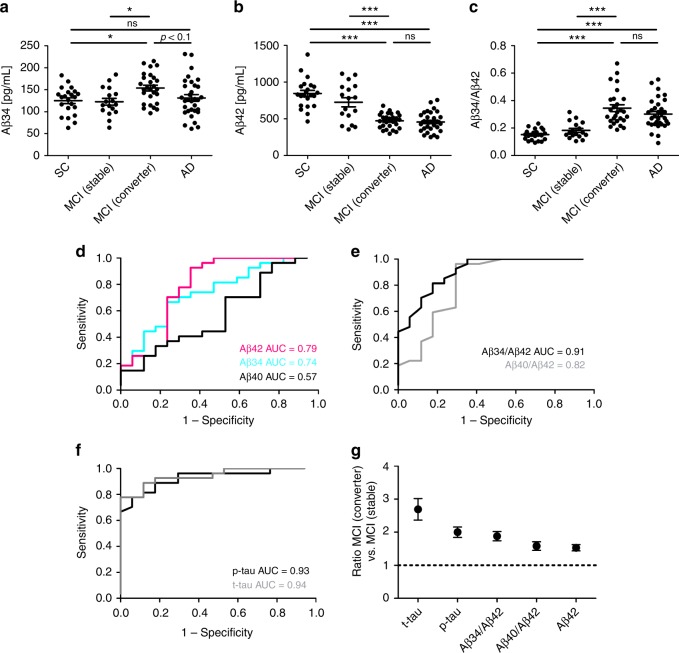
Aβ34 and core biomarkers in the Amsterdam Dementia Cohort. **a**–**c** Analyses of Aβ34 and Aβ42 in human CSF samples. *n* = 22 subjective complaints (SC), *n* = 17 MCI (stable), *n* = 27 MCI (converter), *n* = 32 Alzheimer’s disease (AD). Horizontal lines indicate mean ± s.e.m. The data were analyzed with one-way ANOVAs and Tukey’s post-hoc tests (***p < 0.001, *p < 0.05, ns *p* > 0.05). **a** Aβ34, one-way ANOVA *F*(3, 94) = 3.71, *p* < 0.05, **b** Aβ42, one-way ANOVA *F*(3, 94) = 30.91, *p* < 0.0001, **c** Aβ 34/Aβ42, one-way ANOVA *F*(3, 94) = 21.71, *p* < 0.0001. **d** Receiver operating characteristic (ROC) curves were computed on CSF levels of Aβ34, Aβ40, and Aβ42 in samples from the Amsterdam Dementia Cohort *n* = 17 MCI (stable), *n* = 27 MCI (converter). **e** ROC curves on Aβ40/Aβ42 and Aβ34/Aβ42 ratios from MCI (stable) and MCI (converter)). ROCs were compared using DeLong test: Aβ40/Aβ42 vs. Aβ34/Aβ42 (*p* = 0.0298). **f** ROC curves on p-tau and t-tau from MCI (stable) and MCI (converter)). **g** Comparison of the performance of various molecules measured in CSF, which is based on average MCI (converter) to MCI (stable) ratios in the Amsterdam Dementia Cohort. The MCI (converter) to MCI (stable) ratio of Aβ42 was inverted for better comparison with the other ratios

To explore the associations between Aβ34 and putative changes in BACE1 expression observed in AD^9–11^, we analyzed Aβ34 in 98 human CSF samples from the Amsterdam Dementia Cohort. Samples were collected from 22 people with subjective cognitive complaints (SC), 17 with MCI that remained stable, 27 with MCI that later progressed to AD dementia (i.e., MCI converters), and 32 AD patients. Using our MSD assay, we found significantly elevated Aβ34 levels in the CSF of MCI converters when compared with SC or MCI stable (Fig. 6a). Furthermore, the levels in MCI converters showed a trend for being elevated compared with AD patients (the greater variability is likely due to a larger heterogeneity in this group). CSF-Aβ34 levels from all groups did not associate with age, the genetic risk allele *APOE ε4*, or gender (Supplementary Fig. 5a–c). As expected, there was a decrease in Aβ42 in AD patients and MCI converters compared with the SC and MCI stable group (Fig. 6b). These changes are likely due to the sequestration of Aβ42 into amyloid plaques and indicate that MCI converters and AD patients are typically Aβ+^41^. The Aβ34/Aβ42 ratio is significantly elevated in the MCI converter and AD groups compared with the other groups (Fig. 6c).

To assess whether CSF-Aβ34 could provide important information during the early stages of AD, we tested whether Aβ34 could discriminate between MCI converters vs. non-converters from the Amsterdam Dementia Cohort. Receiver operating characteristic (ROC) curves of the CSF analytes were used to determine the accuracy of distinction between the MCI converters (i.e., prodromal AD) vs. stable patients. The area under the curve (AUC) for Aβ34 alone was slightly smaller than for Aβ42 (Fig. 6d). Interestingly, the Aβ34/Aβ42 ratio (AUC = 0.91) significantly improved the diagnostic accuracy compared with the classical Aβ40/Aβ42 ratio (AUC = 0.82) (Fig. 6e). An optimal cut-off analysis^42^ yielded the best cut-off for an Aβ34/Aβ42 ratio >0.245, where sensitivity was 81.48% and specificity 82.35% (Supplementary Fig. 5d). The improved diagnostic accuracy of the Aβ34/Aβ42 ratio seemed specific for the distinction between MCI converters and stables. Similar AUCs ranging from 0.93 to 0.98 were obtained for Aβ42, Aβ40/Aβ42, and Aβ34/Aβ42 for the distinction between the SC and MCI converters or the SC and AD populations respectively (Supplementary Fig. 5e, f). For the distinction between MCI converters and stable patients, p-tau and t-tau yielded AUCs of 0.93 and 0.94, respectively (Fig. 6f). To assess how Aβ34/Aβ42 compares with the core AD biomarkers^43^, we calculated the ratio between the MCI converter and MCI stable groups (Fig. 6g). Aβ34/Aβ42 ranked third after the core markers, i.e., total- and phosphorylated-tau but before Aβ40/Aβ42 and Aβ42 (Fig. 6g). Overall, these results suggest that using Aβ34, an indicator for Aβ degradation in combination with Aβ42, a core biomarker for Aβ deposition, can improve the accuracy of prediction compared with Aβ40/Aβ42 regarding MCI patients who will convert to dementia vs. non-converters. The Aβ34/Aβ42 ratio could complement but not supplant existing biomarkers, such as CSF levels of total- and phosphorylated-tau. This finding suggests that, at certain stages of Aβ34 elevation in the CSF, the combination of this marker with reduced Aβ42 may indicate a failure in the clearance pathway associated with AD progression.

To test whether changes in the Aβ34/Aβ42 ratio are already detectable in earlier, pre-symptomatic AD, likely even before signs of neuronal injury become evident, we analyzed another 94 human CSF samples from cognitively normal, at-risk individuals from the PREVENT-AD cohort^44^. Individuals enrolled in this study have no diagnosable cognitive dysfunction and are in good general health, but they have a family history of a parent or multiple siblings affected with AD dementia^45–47^. At enrollment, these individuals were on average 10.2 years younger than the age of dementia onset for their earliest-affected relative. Consistent with our earlier Amsterdam Dementia Cohort findings, CSF-Aβ34 levels in the PREVENT-AD samples were not associated with age, *APOE ε4*, or gender (Supplementary Fig. 6a–c). We then tested whether an increased Aβ34/Aβ42 ratio (optimal cut-off Aβ34/Aβ42 > 0.245; Fig. 6e) could also be observed in cognitively normal individuals from the PREVENT-AD cohort. We identified 17 out of 94 individuals (18.09%) with Aβ34/Aβ42 ratios that were above our estimated cut-off.

**Fig. 7:**
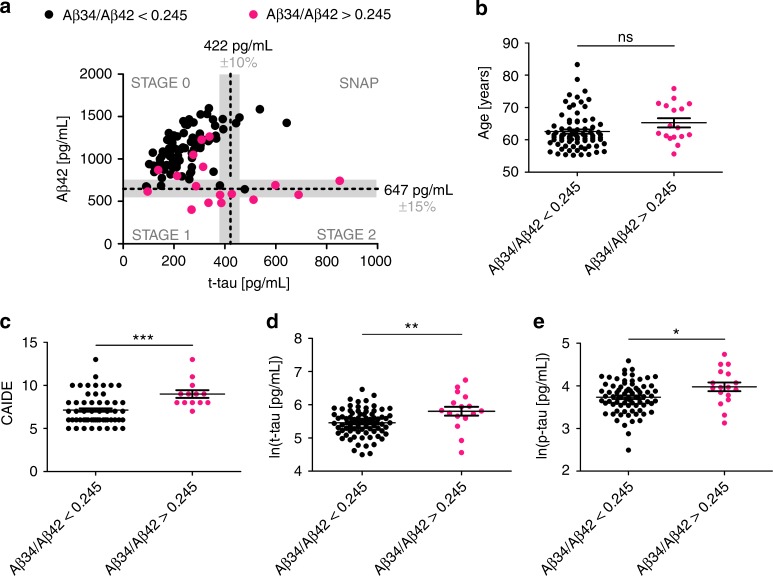
Aβ34, Aβ42, t-tau, and p-tau in PREVENT-AD. **a**–**e** PREVENT-AD Study: analysis of Aβ34, Aβ42, t-tau, and p-tau in CSF samples from cognitively normal individuals at risk for Alzheimer’s disease (*n* = 94). **a** Individuals can be separated into stages of pre-symptomatic AD, based on CSF biomarker assessment (t-tau cut-off > 422 pg/mL^49,50^, Aβ42 cut-off ≤ 647 pg/mL^41^, the gray shaded area indicates common inter-assay variances of the used cut-off values^41,51^). STAGE 0: t-tau and Aβ42 normal; STAGE1: t-tau normal and Aβ42 ≤ 647 pg/mL; STAGE 2: t-tau > 422 pg/mL, and Aβ42 ≤ 647 pg/mL; Suspected-non-AD pathology (SNAP): t-tau > 422 pg/mL and Aβ42 normal. Individuals with Aβ34/Aβ42 ratio above the optimal cut-off calculated in this study (Aβ34/Aβ42 > 0.245) are highlighted in magenta. **b**–**e** Comparison of age, Mann–Whitney *U* = 461.5, *p* = 0.0586 (**b**); Cardiovascular Risk Factors, Aging, and Incidence of Dementia (CAIDE), Mann–Whitney *U* = 184.5, *p* = 0.0004 (**c**); t-tau, unpaired t-test t(92) = 3.027, *p* = 0.0032 (**d**); and P_181_-tau (p-tau), unpaired *t*-test *t*(92) = 2.453, *p* = 0.0168 (**e**); between individuals with Aβ34/Aβ42 ratios above and below optimal cut-off (***p < 0.001, **p < 0.01, *p < 0.05, ns *p* > 0.05). Horizontal lines indicate mean ± s.e.m.

According to current views, the asymptomatic phase of AD is characterized by a sequential appearance of abnormalities, starting with increased cortical Aβ deposition (stage 1), leading to additional signs of neurodegeneration (stage 2; no abnormalities are seen in stage 0)^48^. Figure 7a depicts the Aβ42 and t-tau distribution of the PREVENT-AD samples from individuals at different stages of pre-symptomatic AD, with most individuals remaining in the stage 0 group. The horizontal and vertical dotted lines and shaded areas represent Aβ42^41^ and t-tau cut-off values^49,50^ and their inter-assay variances^51^ (Fig. 7a). The biomarker assessments (validated CSF-Aβ42 cut-off values including the inter-assay variances^41,51^) indicated that 15 PREVENT-AD participants are likely candidates having pre-clinical AD at stages 1 and 2 (Fig. 7a). Out of these, 11 presented with Aβ34/Aβ42 ratios above our cut-off (11/15 = 73.33%) (Fig. 7a). In addition, 6 out of 73 (8.22%) individuals at stage 0 also showed an elevated Aβ34/Aβ42 ratio. Individuals with elevated Aβ34/Aβ42 were not significantly older (Fig. 7b), but they exhibited significantly increased Cardiovascular Risk Factors, Aging, and Incidence of Dementia scores (CAIDE; Fig. 7c), and significantly increased CSF levels of total- and phosphorylated-tau (t-tau, p-tau; Fig. 7d, e).

### CSF-Aβ34 correlates with Aβ clearance in Aβ+ individuals

**Fig. 8:**
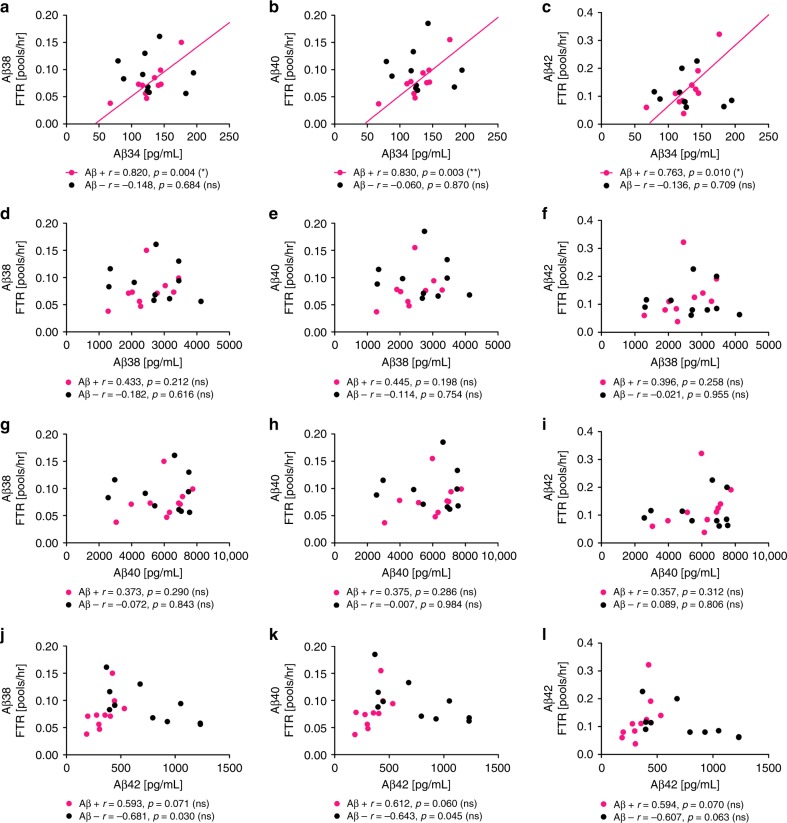
Association between CSF-Aβ34 and Aβ clearance rates. Analysis of Aβ34, Aβ38, Aβ40, and Aβ42 in human CSF with ultra-sensitive assays (Meso Scale Discovery (MSD)). Aβ38, Aβ40, and Aβ42 clearance (fractional turnover rate, FTR) was previously measured using stable isotope labeling kinetic (SILK)^31^. Samples were from *n* = 10 Aβ+ and *n* = 10 Aβ– individuals. Scatterplots of CSF-Aβ34 (**a**–**c**), Aβ38 (**d**–**f**), Aβ40 (**g**–**i**), or Aβ42 (**j**–**l**) with Aβ38 FTR (**a**, **d**, **g**, **j**), Aβ40 FTR (**b**, **e**, **h**, **k**), or Aβ42 FTR (**c**, **f**, **i**, **l**). Pearson correlation coefficients (*r*) were computed to assess the relationship between the variables. The Bonferroni adjusted *p*-values are: **p < 0.003, *p < 0.016, ns = nonsignificant *p* > 0.0125

In MCI and sporadic AD brain tissue, the levels and enzymatic activity of BACE1 are increased^9–11^ and localized to the surroundings of amyloid deposits^12–15^. Above, we presented evidence that Aβ34 (an amyloidolytic product of BACE1) is associated with BACE1-mediated Aβ clearance and is elevated in MCI converters, who show evidence of amyloid plaques (based on their CSF-Aβ42 levels^41^). Thus, we analyzed the CSF concentrations of Aβ34, Aβ38, Aβ40, and Aβ42 in Aβ+ and amyloid-negative (Aβ–) individuals (10 individuals per group; Fig. 8), whose Aβ turnover was previously assessed by SILK^TM^ ^31^. SILK^TM^ data of these 20 individuals were previously reported^31^ and we correlated our results with the published data. The fractional turnover rate (FTR) or true fractional clearance rate^31^, which is associated with the irreversible loss of Aβ38, Aβ40, and Aβ42^52^, showed a significant positive correlation with the CSF-Aβ34 concentrations only in Aβ+ individuals (Fig. 8a–c). Moreover, the CSF concentrations of Aβ38, Aβ40, and Aβ42 showed no significant correlation with Aβ38, Aβ40, or Aβ42 FTRs in our dataset (Fig. 8d–l). Interestingly, the ratio Aβ34/Aβ42 correlated with all three FTRs in Aβ– individuals, whereas the other ratios showed no correlation (Supplementary Fig. 7a–i). Overall, the correlation between Aβ34 and the clearance of the longer Aβ species is consistent with our results from rodent brains and SH-SY5Y cells, suggesting that elevated BACE1 levels in Aβ+ individuals might shift Aβ38, Aβ40, and Aβ42 into the Aβ34 degradation pathway. In conclusion, Aβ34 might serve as a surrogate marker for the overall clearance of Aβ38, Aβ40, and Aβ42 in Aβ+ and the ratio Aβ34/Aβ42 for the overall clearance in Aβ– individuals.

## Discussion

In the AD field, research has traditionally focused on the conversion of APP into Aβ42 peptides leading to pathological amyloid plaque deposition in the brain and cognitive decline during the clinical progression of the disease. In recent years, however, emerging literature now identifies amyloid clearance as an important paradigm to better understand amyloid imbalances in sporadic AD patients.

BACE1 is thought to play a major role in the pathogenesis of AD and several inhibitors have been evaluated in clinical trials for their potential to slow or halt the production of neurotoxic Aβ peptides^8^. In order to attenuate disease progression in individuals with existing amyloid plaques, Aβ production would need to be inhibited by at least 95% since deposition is expected to be fast in Aβ+ individuals, even at lowered rates of newly produced Aβ^53,54^. Recently, current treatment approaches have been challenged based upon reports of disappointing results and adverse effects from BACE inhibitor trials (press release, 25 October 2018, Is there a role for BACE inhibition in Alzheimer’s treatment?, Clinical Trials on Alzheimer’s Disease (CTAD) conference).

BACE1 levels are increased in the AD brain^9–11^, and this could potentially result in increased Aβ production. However, a ~50% reduction in cerebral BACE1 (as reported in BACE1+/− mice and rats) does not alter Aβ38, Aβ40, and Aβ42 levels, suggesting that half the usual amount of BACE1 is sufficient to fully process endogenous APP^32,33,35,36^. Furthermore, increased enzyme levels do not lead to an increased amyloid load in the brains of mice overexpressing human BACE1^55^.

We found that cerebral Aβ34 levels were decreased by about 30% in BACE1+/− mice and by about 40% in wild-type rats, 1 h after intravenous injection of 1 mg/kg MK-8931 (with longer Aβ species unaltered). In the CSF of these MK-8931-treated rats, Aβ34 was the only Aβ species that was significantly decreased in the 1 and 20 mg/kg groups. However, since we administered the inhibitor intravenously, the greatest reduction in Aβ was likely achieved much earlier than in the original study, where rats received the compound orally and Aβ40 was significantly reduced in CSF and cortex at 1 and 3 h^37^. Due to the different routes of administration, our data cannot be directly compared with this study^37^. In contrast to the initial APP cleavage, BACE1 is a limiting factor for the amyloidolytic cleavage of longer Aβ species into Aβ34. Furthermore, our data imply that cerebral BACE1 possesses Aβ-degrading properties in vivo, as consistent with an earlier hypothesis based on in vitro studies^19–21^. The dichotomy between the amyloidogenic and amyloidolytic roles of BACE1 becomes more evident in experimental systems with elevated BACE1 levels. In the present study, for example, we found that an excess of APP favors amyloidogenic Aβ peptide production, but an excess of BACE1 results in increased Aβ degradation to Aβ34. Our characterization of the BACE1-mediated Aβ34 pathway is consistent with previous findings that cerebral BACE1 is not limiting for Aβ production^32,33,35,36^, observations that until now had remained unexplained due to our lack of understanding of amyloidolytic BACE1 activity.

As BACE1-derived Aβ34 can be further degraded by PA-sensitive metalloproteases^21^, it may be classified as a metastable intermediate in the degradation cascade of amyloidogenic peptides. The current findings show that this process is regulated by BACE1 expression such that when BACE1 may become limiting (i.e., when APP is overexpressed), amyloidogenic Aβ peptides are enzymatically degraded by both metalloproteases and BACE1. However, when BACE1 is present in sufficient or excess amounts (i.e., when overexpressed), the Aβ34 pathway is favored. As BACE1 is strongly expressed in neurons, particularly at sites of Aβ production in the brain^14,15^, longer Aβ peptides may be favorably converted into non-toxic Aβ34 at these sites. It is likely that Aβ34 might have a specific biological function and anti-apoptotic actions of this fragment on cultured human cells have been described^21^, however, a more thorough characterization is needed in order to determine its physiological role.

Site-specific mutations in APP were found in rare cases of familial early-onset AD (FEOAD) and processing at the β-cleavage site of APP could be causally linked to increased and decreased risk of AD. For example, β-cleavage of APP carrying the Swedish mutation (KM670/671NL) is increased and causes FEOAD^56^. In contrast, the Icelandic mutation (A673T) protects against AD^57^, primarily by reducing the β-cleavage of APP, as well as modulating Aβ aggregation^58^. In light of our findings, it is plausible that the Icelandic mutation results in reduced Aβ generation, whereas BACE1-mediated Aβ degradation still occurs. Although inhibition of BACE1-mediated cleavage of APP remains an attractive therapeutic approach in AD, it appears that reducing BACE1-mediated generation of pathogenic Aβ peptides alone will not be sufficient to stop plaque growth^53,54^. Furthermore, BACE1 inhibition is likely to affect amyloidolytic cleavage of longer Aβ species into smaller, non-amyloidogenic Aβ34. For this reason, we tested for clearance effects in human CSF and, notably, we found elevated baseline levels of Aβ34 in MCI patients who later progressed to AD. Aβ34 can be detected in human plasma samples^59^, therefore, it would be interesting to test whether its levels in CSF and plasma correlate or whether MCI converters show elevated amounts of plasma-Aβ34. By combining an indicator for Aβ degradation (i.e., Aβ34) and a biomarker for Aβ deposition (i.e., Aβ42), we found that the Aβ34/Aβ42 ratio significantly improved the diagnostic accuracy to distinguish between prodromal AD and stable MCI compared with the classical Aβ40/Aβ42 ratio. However, elevated CSF levels of p-tau and t-tau showed the best distinction between the two MCI groups. An elevated Aβ34/Aβ42 ratio in the prodromal stage of AD could indicate that, at early stages of Aβ plaque formation (i.e., decreased CSF-Aβ42 levels^41^), the increased levels and amyloidolytic activity of BACE1 elicit a defense reaction (i.e., increased generation of Aβ34 to facilitate amyloid clearance). Although BACE1 levels are elevated around fibrillar Aβ^12–15^, the fibrillar conformation is resistant to BACE1 cleavage because of its unique structure^60^ (under the condition of their co-presence in a cellular compartment with a low pH). Aβ42 fibrils are stabilized by hydrophobic clusters in such a way that they do not grant BACE1 access to the Aβ34 cleavage site^60^. Furthermore, since only Aβ42 turnover (and not Aβ38 or Aβ40) is altered when amyloidosis has started^31^, the surplus of Aβ34 could be mainly derived from Aβ42 since its conformation is different from Aβ38 and Aβ40. We speculate that intracellular Aβ42 can adopt a conformation that is favorable for fibril formation, which makes it especially susceptible to BACE1-mediated degradation at the Aβ34 cleavage site.

We hypothesized that the Aβ34/Aβ42 ratio might be potentially useful to monitor pre-symptomatic AD, as changes in the classical biomarkers of AD pathogenesis can already be observed before cognitive symptoms appear^48^. In human samples from the PREVENT-AD cohort (i.e., at-risk individuals without current cognitive impairment), we found that the Aβ34/Aβ42 ratio was elevated, especially in individuals whose biomarker assessment classified them in stages 1 and 2 (signs of cortical Aβ deposition). Overall, few individuals in PREVENT-AD showed reduced CSF-Aβ42 ( ≤ 647 pg/mL^41^). In families with autosomal-dominant AD, reduced CSF-Aβ42 and increased tau in asymptomatic mutation carriers were already detected 10–20 years before the estimated age of onset^61^. At present, members of the PREVENT-AD cohort tend to be several years younger compared with the onset of dementia in their affected relative(s) and, in contrast to mutation carriers, they probably vary substantially in their degree of progression of pre-symptomatic AD. Interestingly, we identified individuals in stage 0 with an elevated Aβ34/Aβ42 ratio. However, our data are cross-sectional and should not be interpreted as representing change over chronological ageing. Therefore, it will be crucial to follow the longitudinal trajectory of Aβ34 in these individuals, in combination with classical CSF measures, since we expect that these biomarker changes are closely related to aging, as previously seen in cognitively normal middle-aged volunteers^62^.

Consistent with the concept of amyloid clearance and deposition mechanisms indicating failed clearance in pre-symptomatic stages of AD, we find that the Aβ34 levels in CSF correlate with the overall clearance rates of Aβ38, Aβ40, and Aβ42 in Aβ+ but not Aβ– individuals. In contrast, the CSF Aβ34/Aβ42 ratios correlate with the overall clearance rates of Aβ38, Aβ40, and Aβ42 in Aβ– but not Aβ+ individuals. Since inter-individual variances in Aβ clearance rates are affected by various factors including age, genetics, or pathological processes^24,31,52,63^, it is rather unlikely that the levels of an individual protein, such as BACE1, could determine the overall Aβ clearance from the brain. However, our findings suggest that under pathological conditions, elevated BACE1 levels in the brain direct a large proportion of Aβ38, Aβ40, and Aβ42 into BACE1-mediated Aβ clearance via the Aβ34 degradation pathway. Given this, Aβ34 might be used as a marker for the overall clearance of these peptides in Aβ+ individuals. We speculate that under non-pathological conditions, a special relationship exists between Aβ34 and Aβ42, which might explain the correlation of their ratio with the overall Aβ clearance. Once Aβ42 gets deposited in plaques, the correlation with clearance is lost, likely due to an altered Aβ42 degradation. Under these circumstances, Aβ34 alone becomes the predictor of clearance rates.

**Fig. 9:**
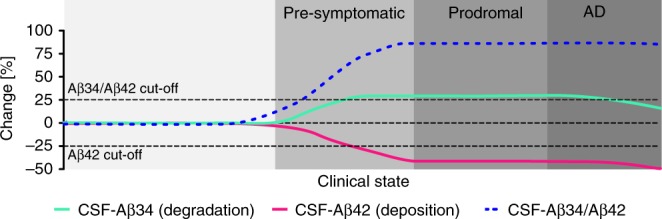
Conceptual model of early changes in AD. Before a clinical diagnosis of Alzheimer’s disease (AD), decades of Aβ peptide deposition lead to plaque formation in pre-symptomatic and prodromal stages of the disease. Incorporating Aβ34 (marker of enzymatic Aβ degradation) with measures of Aβ42 (marker for cerebral Aβ deposition) could complement current biomarker assessments and provide additional information about Aβ turnover

In summary, our results show that, in vivo, BACE1 is limiting for the degradation of longer Aβ peptides into the intermediate Aβ34. The levels of this amyloidolytic fragment are elevated in the CSF of prodromal AD patients (i.e., MCI that progresses to AD). Thus, incorporating Aβ34 as a marker of amyloid clearance with a marker of amyloid deposition (i.e., Aβ42) might complement current CSF measures, especially in clinical intervention trials that aim at a modulation of APP processing (Fig. 9). Ultimately, the present study proposes that enzymatic processes affecting Aβ metabolism are altered in early phases of AD and, accordingly, Aβ34 can be used to monitor Aβ turnover at earlier stages of this devastating disease.

## Methods

### Synthetic Aβ peptides

Synthetic peptides Aβ34, Aβ35, Aβ38, Aβ40, and Aβ42 (PSL, Germany) dissolved in formic acid and vacuum dried in a speed-vac (Thermo) were resuspended (2 mg/mL) in 1% Milli-Q/ammonia water, ultrasonicated (10 min 4 °C), diluted to 1 mg/mL with Milli-Q and ultrasonicated again. Peptide concentrations (ε = 1490 M/cm), integrity, and molecular weight were confirmed by absorption measurements at 280 nm (Synergy H1, BioTek Instruments Inc. plate reader), Coomassie dye stains after performing denaturing polyacrylamide gel electrophoresis (SDS-PAGE), and matrix-assisted laser desorption ionization mass spectrometry (Bruker UltrafleXtreme MALDI-TOF/TOF system in standard reflector-positive mode; samples mixed 1:1 with α-cyanocinnamic acid matrix and applied to ground steel targets using dried-droplet method).

### BACE1-mediated Aβ degradation in vitro

BACE1 from Fc purification^64^ 10 μg/mL (a kind gift from Johan Lundkvist, AstraZeneca) was incubated with synthetic Aβ35, Aβ40, or Aβ42 at 50 μg/mL for 10 min at 37° C in 20 mM (sodium acetate/HCl, pH4.5), directly mixed 1:1 with α-cyanocinnamic acid matrix, and applied to ground steel targets using dried-droplet method.

### MSD assay

Using the ELISA Conversion Kit from MSD (USA), an electrochemiluminescence-based assay was developed (neo-epitope specific Aβ34 and the sulfo-tagged 6E10 (binds to the N-terminus of human Aβ) or the sulfo-tagged 4G8 for rodent samples (binds to the mid domain of Aβ)). High-bind or custom-printed 4-plex plates (using our mab34, 4G8 (Biolegend) for pan-Aβ assay, G2-10 for 1-plex Aβ40 assay, and MSD’s validated mouse monoclonal anti-Aβ38, anti-Aβ40, as well as anti-Aβ42 antibodies, see MSD Aβ peptide V-PLEX) were blocked (MSD 5% Blocker A in PBS) for 1 h at 22 °C and washed three times with PBS-Tween (PBS-T) for 1 min at 22 °C, loaded with SULFO-TAG^TM^ 6E10 or 4G8 detection antibody (diluted to 1× in MSD Diluent 100) and sample or peptide calibrator (in MSD Diluent 35 or cell culture medium), and incubated for 16 h at 4 °C with shaking at 600 rpm. After three washing steps with PBS-T for 1 min at 22 °C, 150 μL 2× MSD read buffer was added per well. All plates were read using an MSD QuickPlex SQ 120 Imager and data analyzed using MSD Workbench® software. Standard curves were fitted using a non-linear four-parameter logistic fit with 1/*y*^*2*^ weighting. The equation is:

1 $$y = b_2 + \frac{{b_1 - b_2}}{{1 + \left( {\frac{x}{{b_3}}} \right)^{b_4}}}$$

*y* = signal, *x* = concentration, *b*_2_ = estimated response at infinite concentration, *b*_1_ = estimated response at Zero concentration, *b*_3_ = mid-range concentration, *b*_4_ = slope factor.

In the WORKBENCH® software, lower limit of detection (LLOD) was determined as the analyte concentration equivalent to the signal that is 2.5× standard deviations (SD) above the back-fit signal of the blank. Assay performance (inter-plate, intra-plate coefficient of variation (CV), LLOD, and upper limit of detection (ULOD)) were assessed using peptide calibrators in MSD diluent 35 (Supplementary Fig. 1e). Spike-and-recovery and linearity-of-dilution assessments for human CSF samples (compared with calibrators in MSD diluent 35) are given in Supplementary Table 1.

### Western blot analysis

Lithium dodecyl sulfate loading buffer (Invitrogen) with 2-Mercaptoethanol (final concentration 5% (*v*/*v*)) was added to samples and the mix was heated to 70 °C for 10 min. Proteins were separated on 4–12% bis/tris gradient gels. Novex® Sharp Pre-Stained Protein Standard (Invitrogen) was used. Peptides/proteins were transferred onto polyvinylidene fluoride membranes (Millipore) by tank blotting (Bio-Rad) at 4 °C. The primary antibodies, anti-actin C4 dilution 1:4000 (Millipore, Catalog #MAB1501), anti-APP ectodomain 22C11 dilution 1:10,000 (Millipore, Catalog #MAB348), anti-BACE1 D10E5 dilution 1:2000 (Cell Signaling, Catalog #mAb5606), anti-sAPPβ dilution 1:2000 (IBL, Catalog #JP18957), and secondary antibodies dilutions 1:10,000 (horseradish peroxidase-conjugated anti-mouse, anti-rabbit, Promega, Catalog #W4021 and # W4011), were used. Most important full-size western blots are displayed in Supplementary Fig. 8. Signals were recorded on ImageQuant LAS 500 or Amersham Imager 600 (GE Healthcare Life Sciences).

### Mouse brain lysates

We complied with all relevant ethical regulations for animal testing and research. Brains were obtained from BACE1−/− and BACE1+/− mice, as well as their wild-type littermates^65^ in accordance with the guidelines of the Institutional Animal Care and Use Committee at the University of Kiel. Frozen mouse brains were thawed on ice, weighed and homogenized in 100 mM Tris-HCl, 150 mM NaCl, 2× complete protease inhibitor cocktail (Roche) using gentleMACS™ M Tubes/Dissociator at 4 °C (Miltenyi Biotech). Triton X-100 was added for a final concentration of 1% and brain homogenates were lysed for 1 h at 4 °C. Lysates were centrifuged at 10,621 × *g* in a microfuge (Eppendorf) at 4 °C for 15 min to remove nuclear fraction. Samples were diluted in the appropriate buffers for protein determination using bicinchoninic acid assay (BCA assay, Pierce) and MSD assays.

### Pharmacological treatment of rats

We complied with all relevant ethical regulations for animal testing and research. Experiments were approved by the McGill Animal Care Ethics Committee. Six to 8 weeks old male Sprague–Dawley rats were housed at the Douglas Mental Health University Institute animal facility and treatments were performed in accordance with the guidelines of the Canadian Council on Animal Care. Rats were intravenously injected with indicated concentrations of MK-8931 (Selleckchem) or vehicle (20% Cyclodextrin) and samples were collected after 1 h of treatment. CSF was collected with the aid of a stereotaxic instrument to appropriately position the head of the rat and samples stored at −80 °C. Brain tissue samples were harvested and immediately preserved on dry ice, later stored at −80 °C. Rat brain lysates were prepared in the same way as mouse brain lysates.

### Plasmids, mutagenesis

Human full-length BACE1 (isoform A) and human full-length APP (isoform APP695, with an N-terminal Myc tag and a C-terminal FLAG tag), in the mammalian expression vector pCEP4, Hygro (Invitrogen) were used for expression. All constructs were verified by DNA sequencing.

### Cell culture and transfection

Human neuroblastoma (SH-SY5Y) cells (DSMZ No.: ACC 209; DSMZ, Braunschweig/ Germany) were cultured in 50% Dulbecco’s modified Eagle’s medium, 50% Hams-F12, 10% fetal bovine serum, 2 mM l-glutamine, 0.5 mM sodium pyruvate, 1× MEM non-essential amino-acid solution in a humidified incubator at 37 °C with 5% CO_2_. Cells were routinely tested for mycoplasma contamination. SH-SY5Y cells were transfected using TransFectin^TM^ according to the manufacturer’s instructions (Bio-Rad) and stable clones were selected using 250 µg/mL Hygromycin B. For experiments, culturing medium without Hygromycin B was used and conditioned for 16 h. Protease inhibitors, were dissolved in Dimethyl sulfoxide (DMSO) at a 1000× concentration and compared with vehicle treatment (DMSO 1:1000). Cells were harvested on ice. Cell culture supernatants were collected, centrifuged for 10 min at 450 × *g* in a microcentrifuge at 4 °C and used for further analysis. Cells were washed once on ice with ice-cold PBS^++^ and lysed in 20 mM Tris-HCl, pH 7.5, 150 mM NaCl, 20 mM 3-[(3-cholamidopropyl) dimethyl-ammonio]-1-propanesulfonate (CHAPS), 2× Complete protease inhibitor (Roche), for 60 min at 4 °C. Cell lysates were centrifuged for 15 min at 10,621 × *g* in a microcentrifuge at 4 °C to remove nuclear fraction.

### CSF samples

We complied with all relevant ethical regulations for work with human participants. The studies were performed in accordance with The Code of Ethics of the World Medical Association (Declaration of Helsinki). The studies were approved by the regional ethics committees. Written informed consent was received from participants prior to inclusion in the studies.

CSF samples for initial assay development were received from the Clinic at the Division of Psychiatry, Zurich. CSF samples from individuals with SILK data^31^ were obtained from the Department of Neurology, Washington University in St. Louis. CSF samples from individuals with different clinical diagnoses were received from the Amsterdam Dementia Cohort and the time interval between CSF collection and assessment of cognition was <24 h^66^. CSF samples from cognitively normal individuals at risk for AD (PREVENT-AD study) were received from the Douglas Mental Health University Institute and the time interval between CSF collection and assessment of cognition was on average 5.6 ± 3.9 (SD) months^44^. The experimentalist was blinded from diagnosis until completion of measurements. Diagnoses of probable AD^67^ or MCI^68^, were made by consensus of a multidisciplinary team according to diagnostic criteria. For the Amsterdam Dementia Cohort, patients who presented with cognitive complaints but were considered as normal after thorough investigation (i.e., criteria for MCI, dementia or any psychiatric or neurological results not fulfilled) were defined as patients with subjective cognitive complaints (SC). Subjects were followed annually and MCI to AD conversion (or MCI that remained stable) was defined based on conversion to AD within 3 years after the CSF collection, and stable as no conversion occurred within 3 years. Lumbar punctures after an overnight fast were performed using the Sprotte 24-gauge atraumatic needle. Samples were aliquoted into propylene cryotubes and stored at −80 °C. Procedures from the BIOMARK-APD consortium of the EU Joint Program in Neurodegenerative Disease were used for sample preparation and measurements^69^. A summary of samples included in the study is given in Supplementary Table 2. CSF t-tau, p-tau, and Aβ42 were measured using INNOTEST ELISA; Fujirebio (formerly Innogenetics).

### Statistical analysis

The statistical evaluation was carried out by GraphPad Prism, SPSS, MedCalc Version 18.2.1, and the indicated statistical tests and algorithms (analysis of variance (ANOVA), *t*-test, Pearson correlation, Mann–Whitney *U*-tests, Spearman correlations, De Long statistics to compare ROC curves^70^). The optimal cut-off (point on the ROC curve with the minimum distance (*d*) to sensitivity = 1 and specificity = 1)^42^ was determined using Pythagoras’ theorem:

2 $$d = \sqrt {(1 - \mathrm{sensitivity})^2 + (1 - \mathrm{specificity})^2}$$

Individual data points, mean and s.e.m. are displayed in the figures. Data were tested for normality, using violation of the Shapiro–Wilk test at *p* < 0.01 as the criterion. Data sets not meeting the normality assumption were analyzed using non-parametric tests (as indicated in the figure legends).

### Reporting summary

Further information on research design is available in the Nature Research Reporting Summary linked to this article.

## Supplementary information

Supplementary Information

Reporting Summary

## Data Availability

Data used in the preparation of this article were obtained from the Pre-symptomatic Evaluation of Novel or Experimental Treatments for Alzheimer’s Disease (PREVENT-AD) program (http://www.prevent-alzheimer.ca) data release 2.0 (30 November 2015, Update: 07 June 2016). Other relevant data are available directly from the authors.
