# Supplementary material for: Aβ34 is a BACE1-derived degradation intermediate associated with amyloid clearance and Alzheimer’s disease progression
Source: Nat Commun. 2019 May 20;10:2240. doi: 10.1038/s41467-019-10152-w (PMC6527709; doi:10.1038/s41467-019-10152-w)
Supplement: Supplementary file 1 — Supplementary Information [file 41467_2019_10152_MOESM1_ESM.pdf]

Supplementary Information

Aβ34 is a BACE1-derived degradation intermediate associated with amyloid clearance and Alzheimer's disease progression

Liebsch et al.

Supplementary Methods

Anti-Aβ34 antibody. To quantify Aβ34 without cross-reactivity from other Aβ species, we generated a high-specificity and high-affinity anti-Aβ34 monoclonal antibody (mab34) directed against Aβ27-34. We complied with all relevant ethical regulations for animal testing and research. Balb/cJ mice were immunized with synthetic Aβ27-34 peptide (from PSL, Germany) conjugated to KLH (Thermo Scientific). Splenocytes were fused to P3x63Ag8.653 myeloma cells (DSMZ No.: ACC 88; DSMZ, Braunschweig/ Germany). Hybridoma cells were cultivated in RPMI 1640, 10% fetal bovine serum (FBS w/o bovine Ig), 50 µM β-Mercaptoethanol, 50 U/ml mu rec.IL-6, 1 x Penicillin-Streptomycin, 1 x L glutamine, and 25 mM (4-(2-hydroxyethyl)-1-piperazineethanesulfonic acid) (HEPES) in a humidified incubator at 37°C with 5% CO_2. Mab34 was purified using protein Protein G Sepharose [1].

Surface Plasmon Resonance (SPR). Using a BIACORE T200 system (GE Healthcare Bio-Sciences AB, Upsala, Sweden; v2.0 Control and v1.0 Evaluation software), SPR experiments were performed at 25°C using filtered (0.2 µm) and degassed HBS-EP+ buffer (10 mM HEPES pH 7.4; 150 mM NaCl; 3 mM EDTA; 0.05% (v/v) Tween-20). Using Biacore's Amine Coupling Kit, Mouse Antibody Capture Kit, and CM5 sensors, an anti-mouse Fc antibody (14 µg/mL in 10 mM sodium acetate pH 5.0) was immobilized to the flow cells (~8000 RU each) as recommended by GE. To assess binding specificity, a variety of Aβ peptides (0 – 2 µM; 4-fold dilution series) were titrated over reference (anti-mouse Fc antibody only) and active (~1000 RU mab34 captured to anti-mouse Fc antibody) surfaces in single-cycle kinetic mode (25 uL/min. x 5 min. association + 10 min. dissociation). Between each sample series, surfaces were regenerated at 50 µL/min. using two 30 sec pulses of Pierce Gentle Ag/Ab Elution Buffer pH 6.6 (Thermo Scientific #21027) as well as 10 mM glycine pH 1.7. To assess binding affinity, Aβ34 peptide titrations (0 – 1.6 µM; 3-fold dilution series) were performed in the similar manner using multi-cycle kinetic mode (25 µL/min. x 10 min. association + 20 min. dissociation). All of the double-referenced data [2] presented are representative of duplicate injections acquired from at least three independent trials. To predict an apparent equilibrium dissociation constant (K_D) for mab34, steady-state binding responses (R_{eq}) in the multi-cycle titrations were averaged near the end of each association phase, plotted as a function of analyte concentration (C), and then subjected to non-linear regression analysis (Steady state affinity model in Biacore evaluation software). Based upon its unique neo-epitope design, mab34 specifically recognizes Aβ34 but not Aβ35, Aβ38, Aβ40, and Aβ42, as analyzed by single-cycle surface plasmon resonance (SPR; Supplementary Figure 2a). Additionally, we tested whether Aβ34 variants could bind to mab34. While the Aβ34 K16N - mab34 interaction demonstrated similar binding kinetics compared to the wild type peptide, Aβ34 G33A failed to interact with the antibody

(Supplementary Figure 2b). Complementary multi-cycle SPR titrations (Supplementary Figure 2c) further demonstrated that the dose-dependent, saturable binding between mab34 and the Aβ34 peptide is a high-affinity interaction (apparent equilibrium dissociation constant, K_D ~40 nM; Supplementary Figure 2d). Overall, these results demonstrate that mab34 binds specifically with nanomolar affinity to the C-terminus of Aβ34 (Supplementary Figure 2e).

Development and characterization of an ultrasensitive Aβ34 assay. We developed an ultra-sensitive multiplexing assay (Meso Scale Discovery (MSD)) for the simultaneous quantification of Aβ34, Aβ38, Aβ40, and Aβ42. Our mab34 and MSD's validated mouse monoclonal anti-Aβ38, anti-Aβ40, as well as anti-Aβ42 antibodies (see MSD Aβ peptide V-PLEX) were spotted as the capture antibodies and we used the SULFO-tagged anti-Aβ N-terminal 6E10 antibody (provided by the manufacturer) for detection. The MSD-based assay yielded excellent intra- and inter-assay variances (CVs below 5%), a lower limit of detection (LLOD) <2.5 pg/mL, a high dynamic range (2.5 – 10,000 pg/mL), and no detectable cross-reactivity with Aβ35, Aβ38, Aβ40, or Aβ42 (Supplementary Figure 3a). Furthermore, we tested whether the different biophysical properties of the Aβ peptides tested had an effect on the response in an electrochemiluminescence-based assay. In a pan-Aβ assay, based on the mouse monoclonal 4G8 capture antibody (recognizing Aβ residues 17-24) all peptides, Aβ34, Aβ35, Aβ38, Aβ40, and Aβ42 yielded similar responses that were statistically not different from one another (Supplementary Figure 3b). For CSF measurements, repeated freeze-thaw cycles differentially affected the measurement of the four different Aβ-species, and the measured concentration of all four Aβ species was reduced after a second thawing cycle (Supplementary Figure 3c-f). These results demonstrate that the effect is less severe for shorter Aβ-species and most prominent for Aβ42. However, we conclude that for a precise assessment of the absolute concentration, Aβ-species are best quantified in CSF samples that were only thawed once. Since CSF samples are thawed at room temperature (see procedures from the BIOMARK-APD consortium that we followed here), these results further support that Aβ34 is neither generated nor degraded directly in CSF [3].

Supplementary Figures

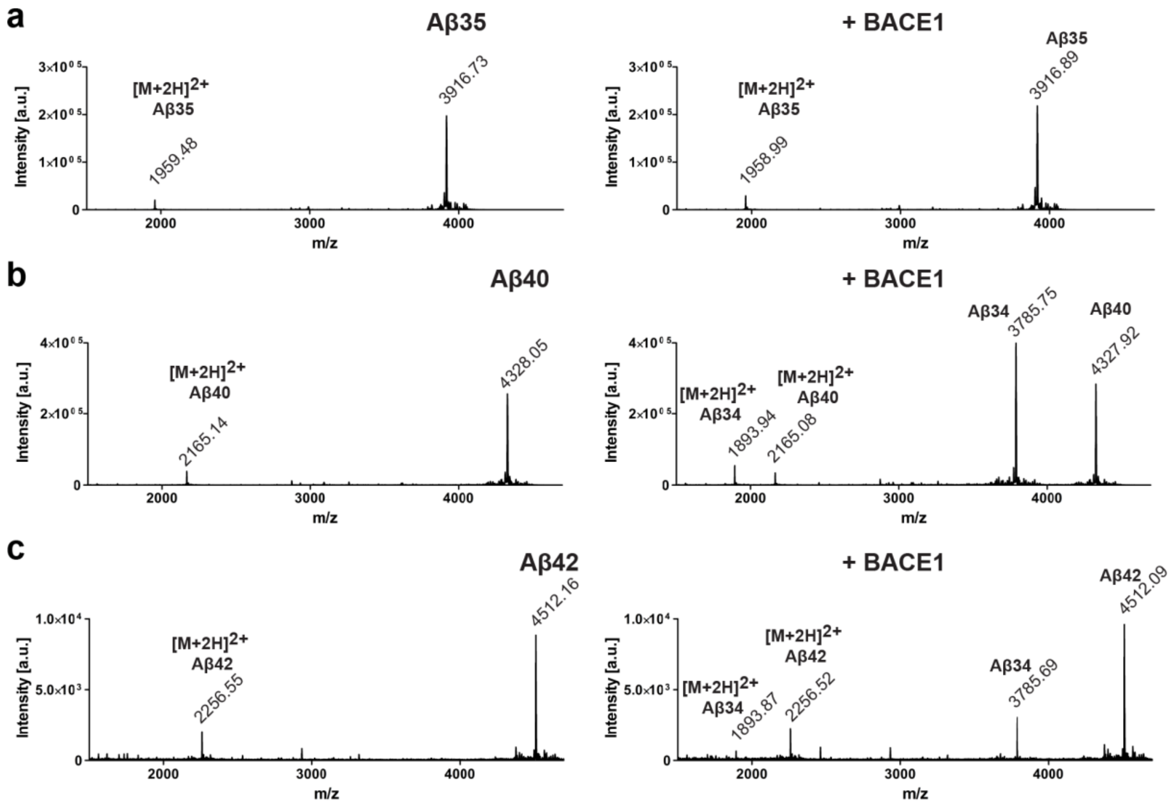

Supplementary Figure 1: BACE1-mediated Aβ degradation *in vitro*

Cleavage of Aβ40 and Aβ42 by BACE1 *in vitro* was analyzed by MALDI-MS. Data were collected from 3 independent experiments. Representative MALDI-MS spectra for Aβ35 (**a**), Aβ40 (**b**), and Aβ42 (**c**), in the absence (left) or presence (right) of BACE1.

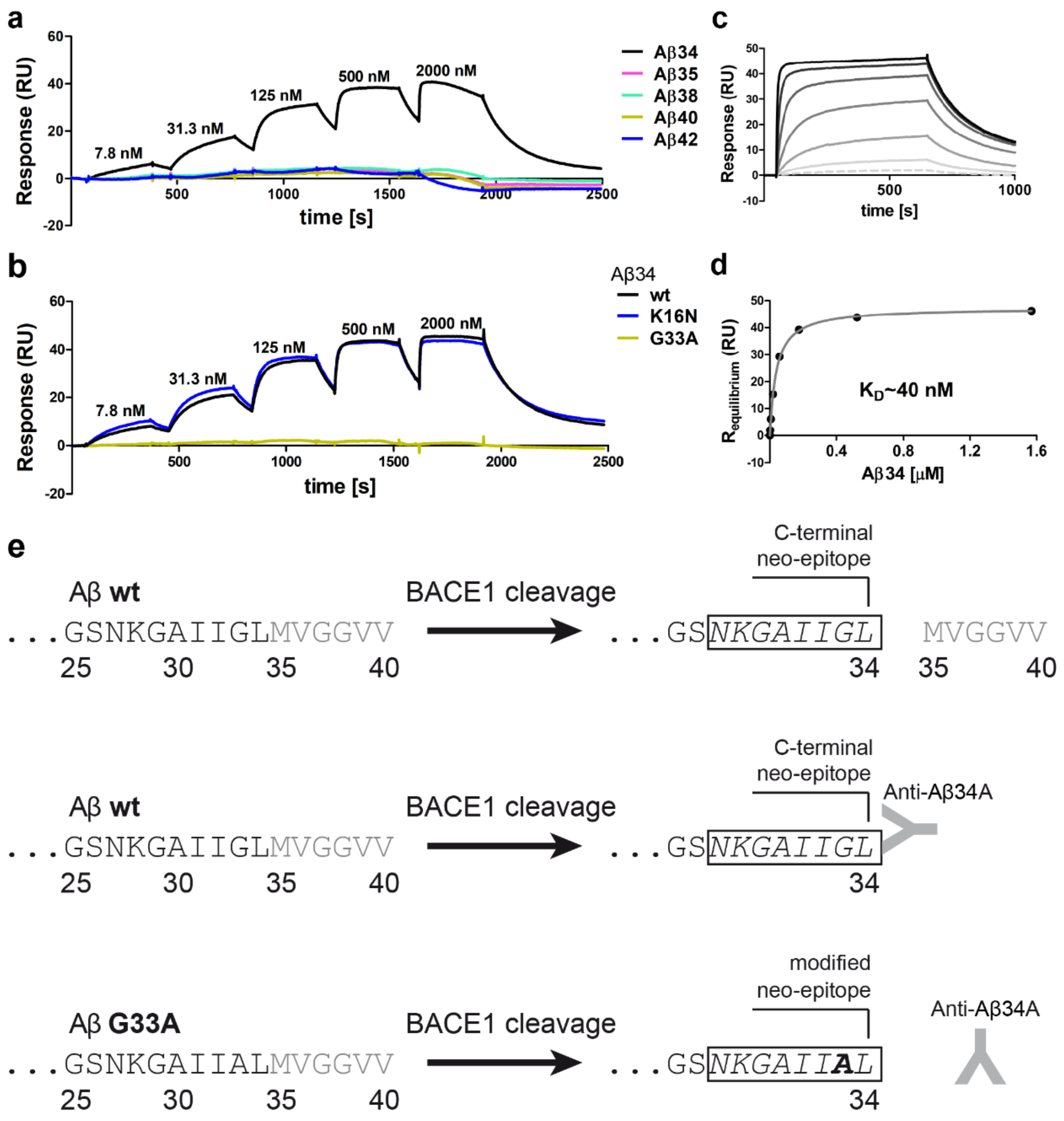

Supplementary Figure 2: High-affinity, neo-epitope antibody against Aβ34

(**a**) Representative single-cycle SPR to demonstrate the specificity and kinetics of synthetic Aβ peptides binding to immobilized anti-Aβ34 antibody (i.e. mab34 captured to anti-Fc sensor). (**b**) Representative single-cycle SPR to demonstrate the specificity and kinetics of synthetic Aβ34 peptide variants binding to mab34 captured to anti-Fc sensor. (**c,d**) Representative multi-cycle SPR demonstrates saturable, dose-dependent binding of Aβ34 peptide (0 – 1.6 μM; 3-fold dilution series, lightest to darkest) to immobilized anti-Aβ34 antibody; steady-state amounts of Aβ34 bound ($R_{equilibrium}$) were plotted as a function of concentration (*symbols*, raw data) and then subjected to non-linear regression analysis (*line*, curve fitting) to determine the apparent equilibrium dissociation constant (K_D). (**e**) Cleavage between L34 and M35, e.g. by BACE1, generates the C-terminal neo-epitope, which is specifically recognized by mab34. The G33A modified neo-epitope is not recognized by mab34.

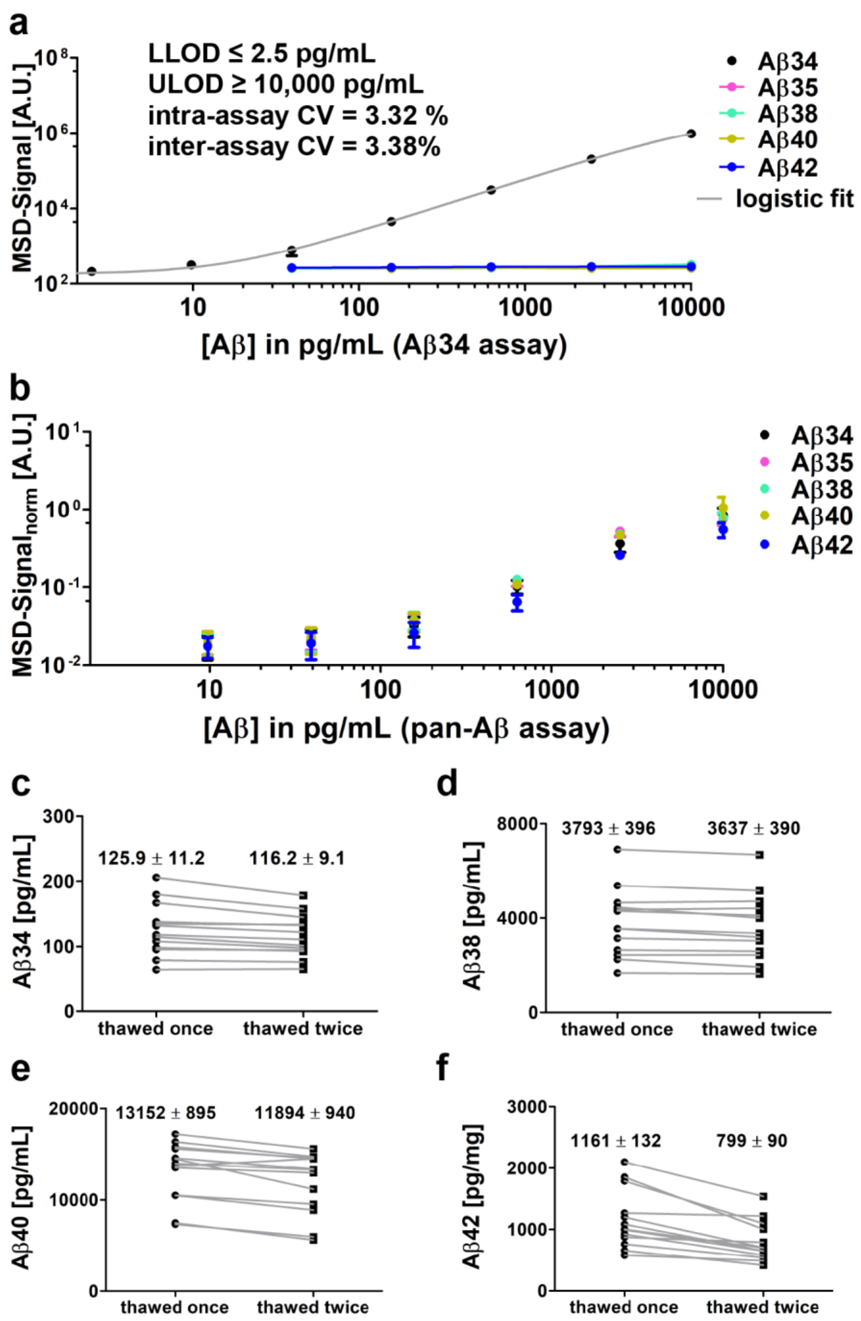

Supplementary Figure 3: Ultrasensitive assay for the quantification of Aβ34

(**a**) Based on the monoclonal neo-epitope specific mab34, an ultra-sensitive, electrochemiluminescence-based immunoassay was developed using MSD technology. The assay was specific for Aβ34 without any significant cross-reactivity against Aβ35, Aβ38, Aβ40, or Aβ42 (n=3 independent experiments, data points for Aβ35, Aβ38, and Aβ40 are overlaid by Aβ42). Measures of assay performance are summarized in the top left corner (n=7 independent experiments). (**b**) Based on the monoclonal anti-Aβ mid-domain (residues 17-24) antibody 4G8, we tested the response of Aβ34, Aβ35, Aβ38, Aβ40, or Aβ42 (n=4 independent experiments) in a pan- Aβ electrochemiluminescence-based assay. Data were analyzed with a 2-WAY ANOVA. While Aβ concentration showed a significant effect $F(5,91) = 57.98$ $p < 0.0001$, there was neither a significant effect for Aβ species $F(4,91) = 1.57$ $p = 0.19$ nor a

significant interaction $F(20,91) = 0.73$, $p = 0.78$. (**c-f**) Using custom-printed MSD 4-plex plates (for Aβ34, Aβ38, Aβ40, and Aβ42), the effect of thawing cycles on the Aβ quantification was tested. Measured concentrations of Aβ34 (**c**), Aβ38 (**d**), Aβ40 (**e**), and Aβ42 (**f**) from CSF samples that were thawed only once or thawed twice (n=15 CSF samples). The mean concentration ± s.e.m. is displayed above the data points. Horizontal lines indicate mean ± s.e.m.

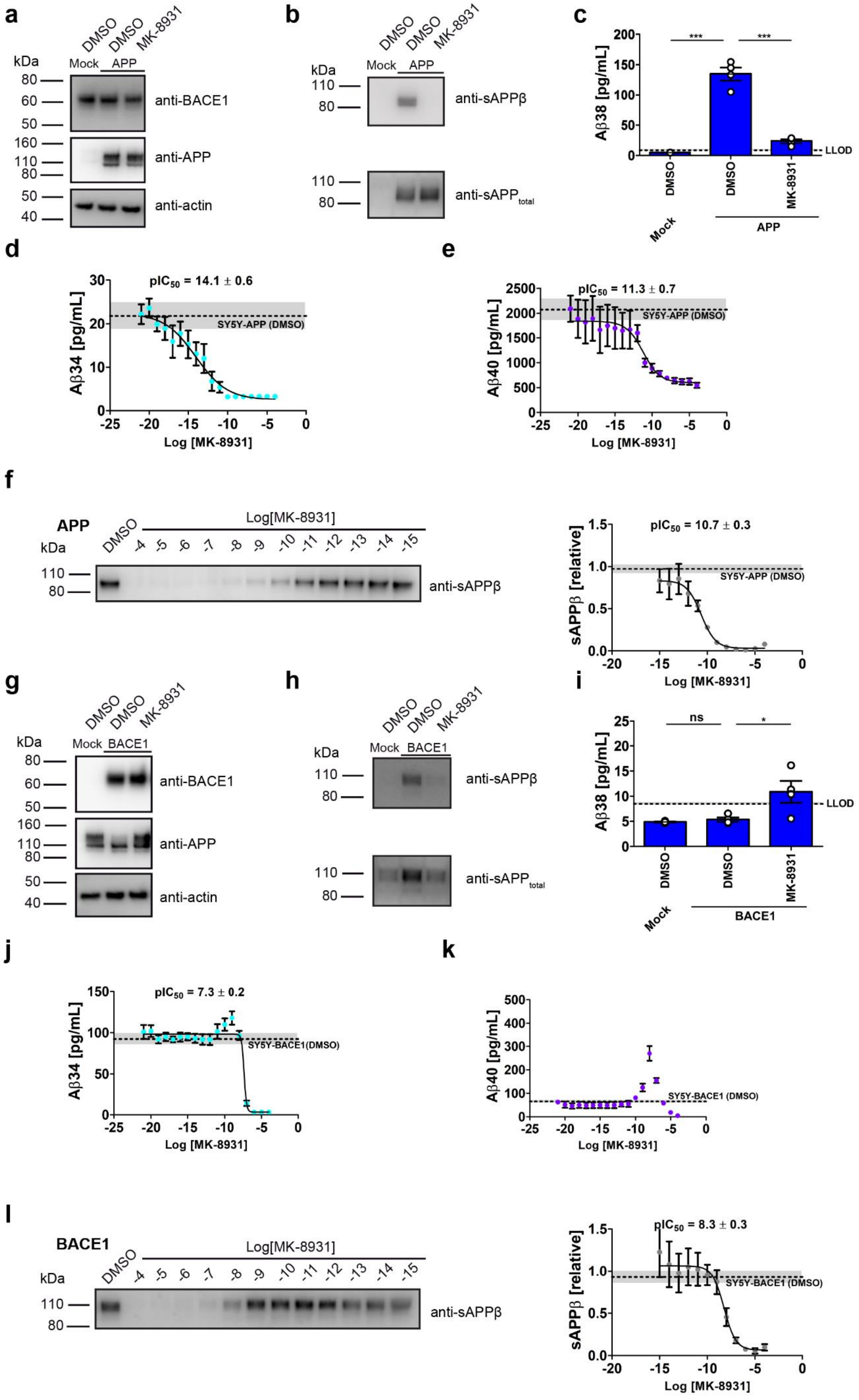
a
DMSO
DMSO
MK-8931
kDa
Mock
APP
80
60
50
160
110
80
50
40
anti-BACE1
anti-APP
anti-actin
b
DMSO
DMSO
MK-8931
kDa
Mock
APP
110
80
anti-sAPPβ
110
80
anti-sAPPtotal
c
Aβ38 [pg/mL]
200
150
100
50
0
***
***
LLOD
DMSO
DMSO
MK-8931
Mock
APP
d
pIC50 = 14.1 ± 0.6
Aβ34 [pg/mL]
30
20
10
0
SY5Y-APP (DMSO)
-25
-20
-15
-10
-5
0
Log [MK-8931]
e
pIC50 = 11.3 ± 0.7
Aβ40 [pg/mL]
2500
2000
1500
1000
500
0
SY5Y-APP (DMSO)
-25
-20
-15
-10
-5
0
Log [MK-8931]
f
APP
DMSO
Log[MK-8931]
kDa
-4
-5
-6
-7
-8
-9
-10
-11
-12
-13
-14
-15
110
80
anti-sAPPβ
pIC50 = 10.7 ± 0.3
sAPPβ [relative]
1.5
1.0
0.5
0.0
SY5Y-APP (DMSO)
-20
-15
-10
-5
0
Log [MK-8931]
g
DMSO
DMSO
MK-8931
kDa
Mock
BACE1
80
60
50
160
110
80
50
40
anti-BACE1
anti-APP
anti-actin
h
DMSO
DMSO
MK-8931
kDa
Mock
BACE1
110
80
anti-sAPPβ
110
80
anti-sAPPtotal
i
Aβ38 [pg/mL]
25
20
15
10
5
0
ns
*
LLOD
DMSO
DMSO
MK-8931
Mock
BACE1
j
pIC50 = 7.3 ± 0.2
Aβ34 [pg/mL]
150
100
50
0
SY5Y-BACE1(DMSO)
-25
-20
-15
-10
-5
0
Log [MK-8931]
k
Aβ40 [pg/mL]
500
400
300
200
100
0
SY5Y-BACE1 (DMSO)
-25
-20
-15
-10
-5
0
Log [MK-8931]
l
BACE1
DMSO
Log[MK-8931]
kDa
-4
-5
-6
-7
-8
-9
-10
-11
-12
-13
-14
-15
110
80
anti-sAPPβ
pIC50 = 8.3 ± 0.3
sAPPβ [relative]
1.5
1.0
0.5
0.0
SY5Y-BACE1 (DMSO)
-20
-15
-10
-5
0
Log [MK-8931]

Supplementary Figure 4: BACE1 inhibition at high substrate or enzyme levels

Representative Western blots for the examination of APP, BACE1, sAPPβ, and $sAPP_{total}$ in APP (**a-f**) or BACE1 (**g-l**) overexpressing SH-SY5Y cells treated with vehicle (DMSO) or BACE1 inhibitor (MK-8931). (**c** and **i**) MSD multiplexing to quantify the absolute amounts of Aβ38. Data were collected from 4 independent experiments. Bars and error bars indicate mean ± s.e.m. (**c,i**) Data were analyzed with 1-WAY ANOVAs and Tukey's post-hoc tests were performed for pairwise comparisons. (**c**) Aβ38, $F(2,9)=118.4$, $p < 0.0001$, (**i**) Aβ38, $F(2,9)=6.8$, $p < 0.05$. (**d-f,j-l**) Quantification of relative amounts of sAPPβ, including representative Western blots (**f,l**), and absolute amounts of Aβ34 (**d,j**) and Aβ40 (**e,k**). Data were collected from 4 independent experiments. Circles and error bars or dashed line with grey shaded area indicate mean ± s.e.m. Dose-inhibition curves were fit with variable slope equations (four parameter model). Above graph $-\log_{10}(IC_{50}) = pIC_{50}$ ± s.e.m are displayed.

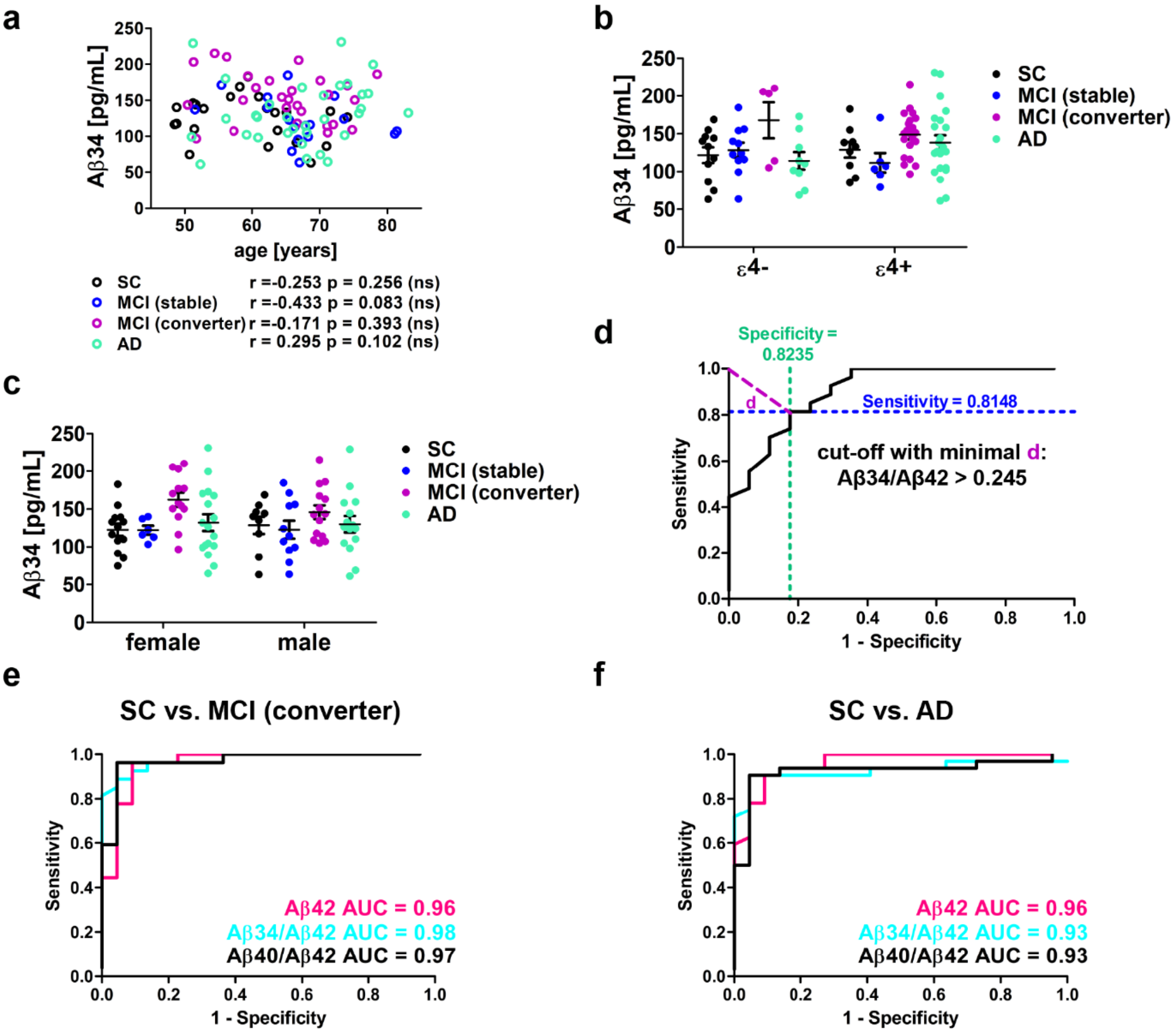

Supplementary Figure 5: CSF-Aβ34 in the Amsterdam Dementia Cohort

(**a**) Scatterplot of Aβ34 with age. Spearman correlation coefficients (r) were computed to assess the relationship between the variables. (**b,c**) The effect of ε4 (**b**) and gender (**c**) on CSF- Aβ34. The data were analyzed with 2-WAY ANOVAs, (**b**) ε4 $F(1,86) = 0.02$, $p=0.89$; diagnosis $F(3,86) = 3.70$, $p=0.02$; interaction $F(3,86) = 1.54$, $p=0.21$ (**c**) gender $F(1,90) = 0.15$, $p=0.70$; diagnosis $F(3,90) = 3.58$, $p=0.02$; interaction $F(3,90) = 0.41$, $p=0.74$. Horizontal lines indicate mean ± s.e.m. (**d**) The optimal cut-off (point on the ROC curve with the minimum distance (d) to sensitivity = 1 and specificity = 1) was determined using Pythagoras' theorem and yielded the best cut-off for a ratio > 0.245, with a sensitivity of 0.8148 and a specificity of 0.8235. (**e,f**) ROC curves were computed on CSF levels of Aβ42, Aβ34/Aβ42, Aβ40, and Aβ40/Aβ42 in samples from n=27 MCI (converter) and n= 22 SC (**e**) and n=32 AD and n= 22 SC (**f**).

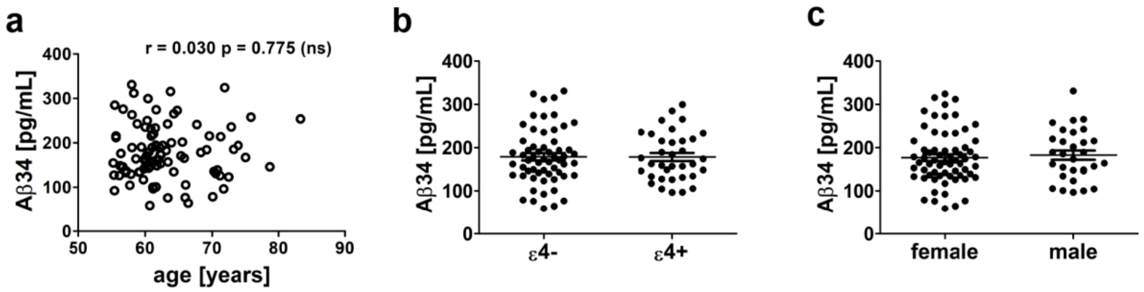

Supplementary Figure 6: CSF-Aβ34 in PREVENT-AD

(**a**) Scatterplot of Aβ34 with age. n=94 CSF samples. Spearman correlation coefficient (r) was computed to assess the relationship between the variables (two-tailed p-value). (**b,c**) The effect of ε4 (**b**) and gender (**c**) on CSF- Aβ34. The data were analyzed with unpaired two-tailed t-tests, (**c**) ε4 $t(92) = 0.02$, $p = 0.98$; (**d**) gender $t(92) = 0.43$, $p = 0.67$. Horizontal lines indicate mean ± s.e.m.

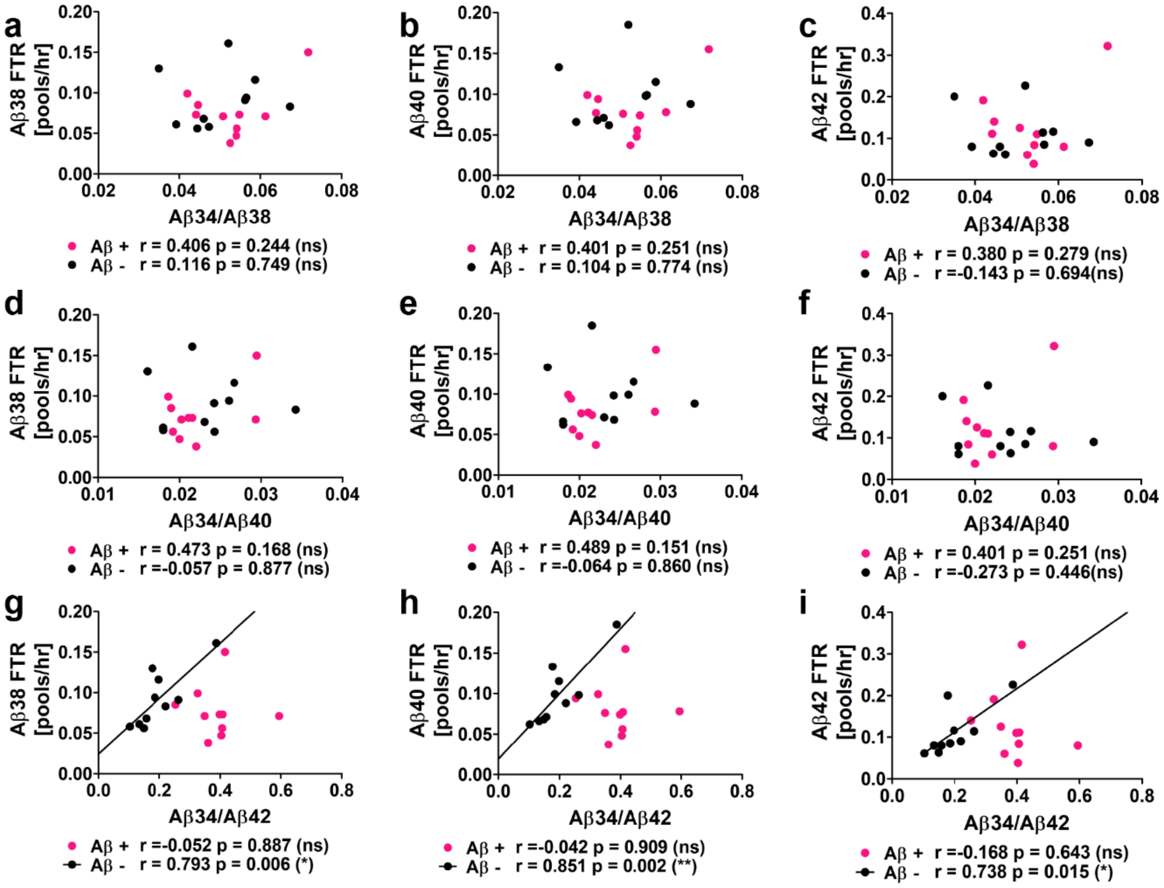

Supplementary Figure 7: Association between CSF-Aβ34/Aβ42 and Aβ clearance

Analysis of Aβ34, Aβ38, Aβ40, and Aβ42 in human CSF with ultra-sensitive assays (Meso Scale Discovery (MSD)). Aβ38, Aβ40, and Aβ42 clearance (fractional turnover rate, FTR) was previously measured using stable isotope labeling kinetic (SILK) [39]. Samples were from n=10 Aβ+ and n=10 Aβ- individuals. Scatterplots of CSF-Aβ34/Aβ38 (**a-c**), Aβ34/Aβ40 (**d-f**), or Aβ34/Aβ42 (**g-i**) with Aβ38 FTR (**a,d,g**), Aβ40 FTR (**b,e,h**), or Aβ42 FTR (**c,f,i**). Pearson correlation coefficients (r) were computed to assess the relationship between the variables. The Bonferroni adjusted p-values are: $^{**}p < 0.003$, $^{*}p < 0.016$, ns = non-significant $p > 0.0125$

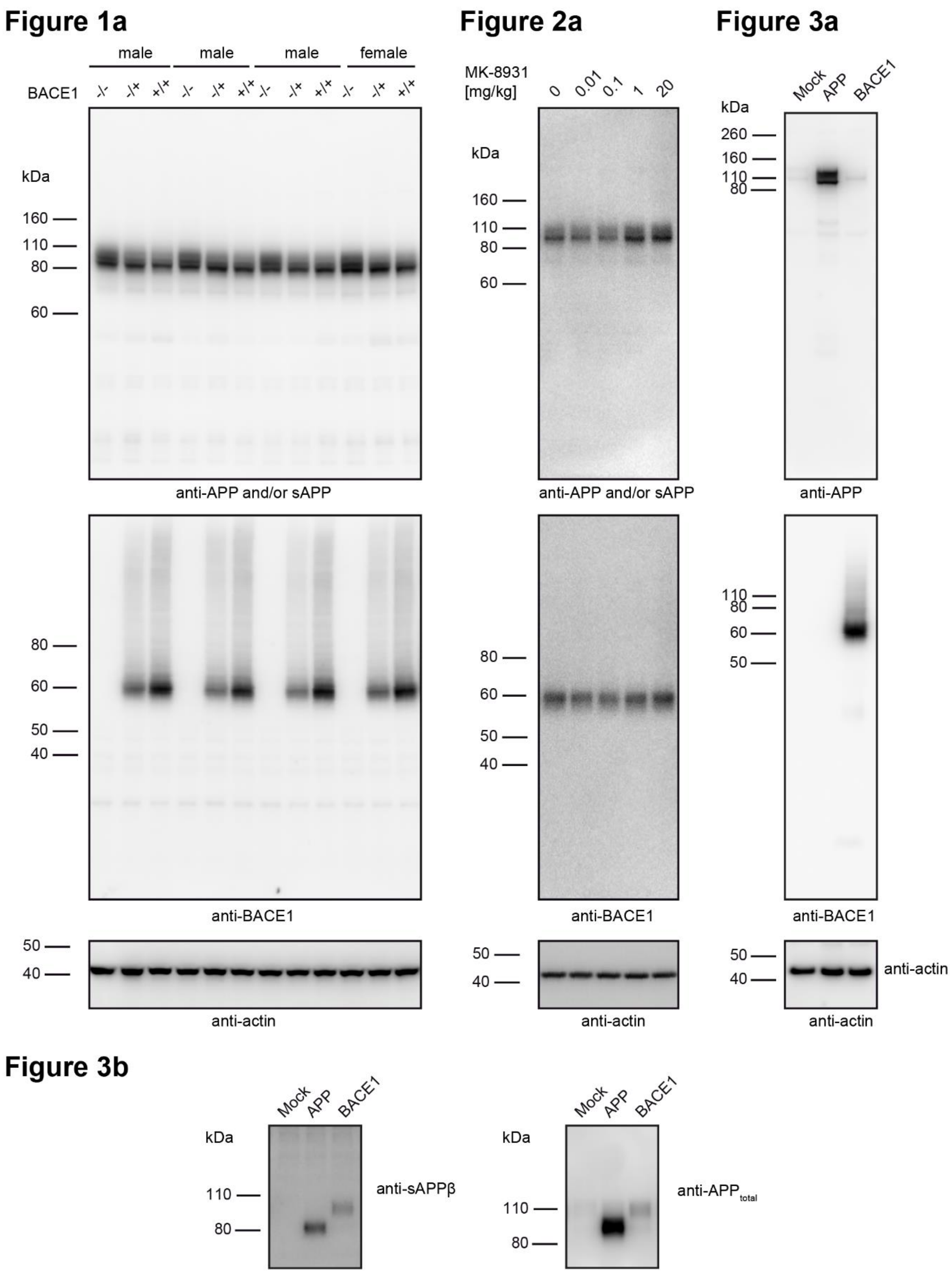

Supplementary Figure 8: Full-size Western blots

Full-size images of Western blots shown in Figures 1, 2, and 3.

Supplementary Tables

Supplementary Table 1. Spike-and-recovery and linearity-of-dilution assessments for human CSF-Aβ34 with MSD

	n	mean Recovery [%] (SD)
neat	4	100
1:2	4	115 (2)
1:4	4	114 (1)
1:8	4	105 (2)
Spike low (1 ng/mL)	4	81 (3)
Spike high (3 ng/mL)	4	85 (3)

Supplementary Table 2. Number and age of Individuals in the Separate Studies

	cognitively normal	SC	MCI	AD
Amsterdam Dementia Cohort (n=98)		n=22	n=44	n=32
female		13/22	19/44	17/32
ε4-carrier (%)		45 (two missing values)	63 (one missing value)	71 (one missing value)
Cognition, Mini-Mental State Examination score (SD)		27.7 (1.4)	26.5 (2.1)	23.3 (4.9)
Age in years (SD)		59.5 (8.1)	67 (8.1)	65.3 (7.7)
PREVENT-AD (n=94)	n=94			
female	64/94			
ε4-carrier (%)	37			
Cognition, Montreal Cognitive Assessment score (SD)	27.8 (1.6)			
Age in years (SD)	63.1 (5.8)			

Supplementary References

1 Ida, N. *et al.* Analysis of heterogeneous A4 peptides in human cerebrospinal fluid and blood by a newly developed sensitive Western blot assay. *The Journal of biological chemistry* **271**, 22908-22914 (1996).

2 Myszka, D. G. Improving biosensor analysis. *J Mol Recognit* **12**, 279-284, doi:10.1002/(SICI)1099-1352(199909/10)12:5<279::AID-JMR473>3.0.CO;2-3 (1999).

3 Portelius, E. *et al.* Ex vivo (18)O-labeling mass spectrometry identifies a peripheral amyloid beta clearance pathway. *Mol Neurodegener* **12**, 18, doi:10.1186/s13024-017-0152-5 (2017).
